# Systemic inflammation index, disease severity, and mortality in patients with COVID-19: a systematic review and meta-analysis

**DOI:** 10.3389/fimmu.2023.1212998

**Published:** 2023-06-21

**Authors:** Arduino A. Mangoni, Angelo Zinellu

**Affiliations:** ^1^Discipline of Clinical Pharmacology, College of Medicine and Public Health, Flinders University, Adelaide, SA, Australia; ^2^Department of Clinical Pharmacology, Flinders Medical Centre, Southern Adelaide Local Health Network, Adelaide, SA, Australia; ^3^Department of Biomedical Sciences, University of Sassari, Sassari, Italy

**Keywords:** systemic inflammation index, risk stratification, COVID-19, disease severity, mortality, biomarkers, inflammation

## Abstract

**Introduction:**

An excessive systemic pro-inflammatory state increases the risk of severe disease and mortality in patients with coronavirus disease 2019 (COVID-19). However, there is uncertainty regarding whether specific biomarkers of inflammation can enhance risk stratification in this group. We conducted a systematic review and meta-analysis to investigate an emerging biomarker of systemic inflammation derived from routine hematological parameters, the systemic inflammation index (SII), in COVID-19 patients with different disease severity and survival status.

**Methods:**

A systematic literature search was conducted in PubMed, Web of Science, and Scopus, between the 1^st^ of December 2019 and the 15^th^ of March 2023. Risk of bias and certainty of evidence were assessed using the Joanna Briggs Institute Critical Appraisal Checklist and the Grades of Recommendation, Assessment, Development and Evaluation, respectively (PROSPERO registration number: CRD42023420517).

**Results:**

In 39 studies, patients with a severe disease or non-survivor status had significantly higher SII values on admission compared to patients with a non-severe disease or survivor status (standard mean difference (SMD)=0.91, 95% CI 0.75 to 1.06, p<0.001; moderate certainty of evidence). The SII was also significantly associated with the risk of severe disease or death in 10 studies reporting odds ratios (1.007, 95% CI 1.001 to 1.014, p=0.032; very low certainty of evidence) and in six studies reporting hazard ratios (1.99, 95% CI 1.01 to 3.92, p=0.047; very low certainty of evidence). Pooled sensitivity, specificity, and area under the curve for severe disease or mortality were 0.71 (95% CI 0.67 to 0.75), 0.71 (95% CI 0.64 to 0.77), and 0.77 (95% CI 0.73 to 0.80), respectively. In meta-regression, significant correlations were observed between the SMD and albumin, lactate dehydrogenase, creatinine, and D-dimer.

**Discussion:**

Our systematic review and meta-analysis has shown that the SII on admission is significantly associated with severe disease and mortality in patients with COVID-19. Therefore, this inflammatory biomarker derived from routine haematological parameters can be helpful for early risk stratification in this group.

**Systematic review registration:**

https://www.crd.york.ac.uk/PROSPERO, identifier CRD42023420517.

## Introduction

Coronavirus disease 2019 (COVID-19) is characterized, particularly in severe cases, by a state of excessive systemic inflammation which, in turn, promotes the dysregulation of specific pathways within the immune, hemostasis and coagulation systems (1–9). Such abnormalities are critical in disrupting several molecular and cellular homeostatic mechanisms, favoring toxicity and dysfunction in different organs and systems (10–12).

Several circulating molecules have been investigated in the quest for markers of excessive inflammatory response to guide early risk stratification and management in patients with COVID-19, including C-reactive protein, pre-albumin, albumin, lactate dehydrogenase, hydroxybutyrate dehydrogenase, and D-dimer (8, 13–15). At the same time, abnormalities in the count of specific blood cell types during the early stages of COVID-19, e.g., neutrophilia, lymphopenia, and thrombocytopenia, and associated haematological indexes, particularly the neutrophil-to-lymphocyte ratio (NLR), have also been shown to be associated with excessive inflammation and to predict severe disease and mortality in this patient group (16–19). Another haematological index, the systemic inflammation index (SII, calculated using the following formula: (neutrophils x platelets)/lymphocytes), investigated for the first time in 2014 in cancer patients (20), has been shown to have a superior predictive capacity for adverse outcomes when compared to other haematological indexes, including the NLR, in patients with COVID-19 (21).

Given the rapidly evolving clinical scenario since the beginning of the COVID-19 pandemic, with the occurrence of novel variants of the causative agent, the severe acute respiratory syndrome coronavirus 2 (SARS-CoV-2), the introduction of anti-inflammatory and immunomodulatory treatments, and the roll-out of vaccination programs, we conducted a systematic review and meta-analysis of the association between the SII and severe disease and mortality in patients with COVID-19. We speculated that patients with a severe disease or succumbing from the disease had higher SII values than patients with a non-severe disease or survivor status. Where possible, we performed meta-regression and subgroup analyses to investigate possible associations between the effect size of the between-group differences in the SII and pre-specified study and patient characteristics.

## Methods

### Literature search and study selection

We conducted a systematic literature search for articles published in the electronic databases PubMed, Web of Science, and Scopus, between the 1^st^ of December 2019 and the 15^th^ of March 2023, using the following terms (and their combination): “SII” or “Systemic Inflammation Index” and “COVID 19” or “2019-nCoV” or “SARS-CoV-2” or “coronavirus disease 2019”. We also hand-searched the reference lists of individual articles to identify additional studies. The criteria for inclusion were: (a) investigation of COVID-19 patients with different disease severity or survival status; (b) reporting of the SII as a continuous variable in COVID-19 patients; (c) reporting of odds ratio (OR) or hazard ratio (HR) with 95% confidence intervals (CIs) for measures of severe disease and/or survival using multivariate analysis; (d) reporting of the prognostic accuracy of the SII using the area under the receiver operating characteristic curve (AUROC) with 95% CIs; (e) full-text available, and (f) English language used. Abstracts and, if relevant, full articles were independently reviewed by two investigators, with a third involved in case of disagreement.

Data extracted from each study included age, sex, year of publication, study design (prospective or retrospective), geographic area where the study was performed, sample size, the clinical endpoint studied (measures of disease severity and/or mortality), markers of inflammation (albumin, lactate dehydrogenase, C-reactive protein, ferritin), markers of renal function (creatinine), markers of coagulation (D-dimer), a history of diabetes, hypertension, and cardiovascular disease, the area under the receiver operating characteristic curve (AUROC) with 95% CIs, sensitivity, specificity, and cut-off values used for the SII. True positive (TP), false positive (FP), false negative (FN), and true negative (TN) values were either extracted or calculated by generating 2 × 2 tables from each study. Sensitivity and specificity were derived from the following formulas: Sensitivity = TP/(TP + FN); Specificity = TN/(FP + TN).

We assessed the risk of bias using the Joanna Briggs Institute Critical Appraisal Checklist for case-control studies. Studies addressing ≥75% of the checklist items were considered as having a low risk (22). The certainty of evidence was assessed using the Grades of Recommendation, Assessment, Development and Evaluation (GRADE) Working Group system (23). The study was conducted per the PRISMA 2020 statement on reporting systematic reviews and meta-analyses (**Supplementary Tables 1, 2**) (24). The protocol was registered in the International Prospective Register of Systematic Reviews (PROSPERO, CRD42023420517).

### Statistical analysis

We generated forest plots of continuous variables, using standardized mean differences (SMDs), to assess differences in the SII between patients with a non-severe disease or survivor status and those with a severe disease or non-survivor status (p<0.05 for statistical significance). Data regarding the associations between the SII and disease severity and mortality, expressed either as odds ratio (OR) or hazard ratio (HR), adjusted for confounding variables, and 95% CIs were also extracted. The ORs were then transformed into log ORs, and the standard error was calculated based on the corresponding 95% CI. We assessed heterogeneity using the Q statistic (p<0.10 for statistical significance). A random-effect model was used in the presence of significant heterogeneity (25). Sensitivity analyses were conducted to investigate the effect of sequentially removing individual studies on the overall risk estimate (26). The presence of publication bias was assessed by investigating the associations between the study size and the magnitude of effect using the Begg’s adjusted rank correlation test and the Egger’s regression asymmetry test (p<0.05 for statistical significance) (27, 28), and the Duval and Tweedie “trim-and-fill” procedure (29). Univariate meta-regression analyses were conducted to investigate associations between the effect size and the following parameters: age, sex, year of publication, study design, sample size, albumin, lactate dehydrogenase, creatinine, D-dimer, C-reactive protein, ferritin, and history of diabetes, hypertension, and cardiovascular disease. Sub-group analyses were also conducted to investigate possible differences in effect size according to specific endpoint studied (disease severity vs. mortality) and the continent where the study was conducted.

We used relevant commands (metandi, midas, mylabels) to evaluate the performance of the SII in predicting severe disease and mortality. A summary receiver operating characteristic (SROC) curve was generated using the hierarchical summary receiving operator characteristic (HSROC) model (30). This was complemented by empirical Bayes estimates that closely agree with those of a full Bayesian analysis. The pooled sensitivity and specificity values were calculated, and the corresponding forest plot was generated. The HSROC model also allowed controlling for heterogeneity across the studies, as determined by i) the correlation coefficient between logit transformed sensitivity and specificity [Corr(logits)] in the HSROC analysis using a bivariate model (31) and ii) the asymmetry parameter β. A positive correlation coefficient (>0) and a β value with p<0.05 indicated the presence of heterogeneity between studies (30, 32). We also explored heterogeneity across studies through visually examining the HSROC curve and using a bivariate boxplot (by midas). The Cook’s distance measurement was performed to estimate the influence of each data point on the overall results of the meta-analysis and identify outliers (33). Publication bias was assessed using the Deeks’ method (34). The relationship between the prior probability, the likelihood ratio, and the posterior test probability was assessed using the Fagan’s Nomogram plot (35). All analyses were performed using Stata 14 (StataCorp LLC, College Station, TX, USA) except for those involving prognostic accuracy, performed using MedCalc for Windows, version 20.109 bit (MedCalc Software, Ostend, Belgium).

## Results

### Study selection

**Figure 1:**
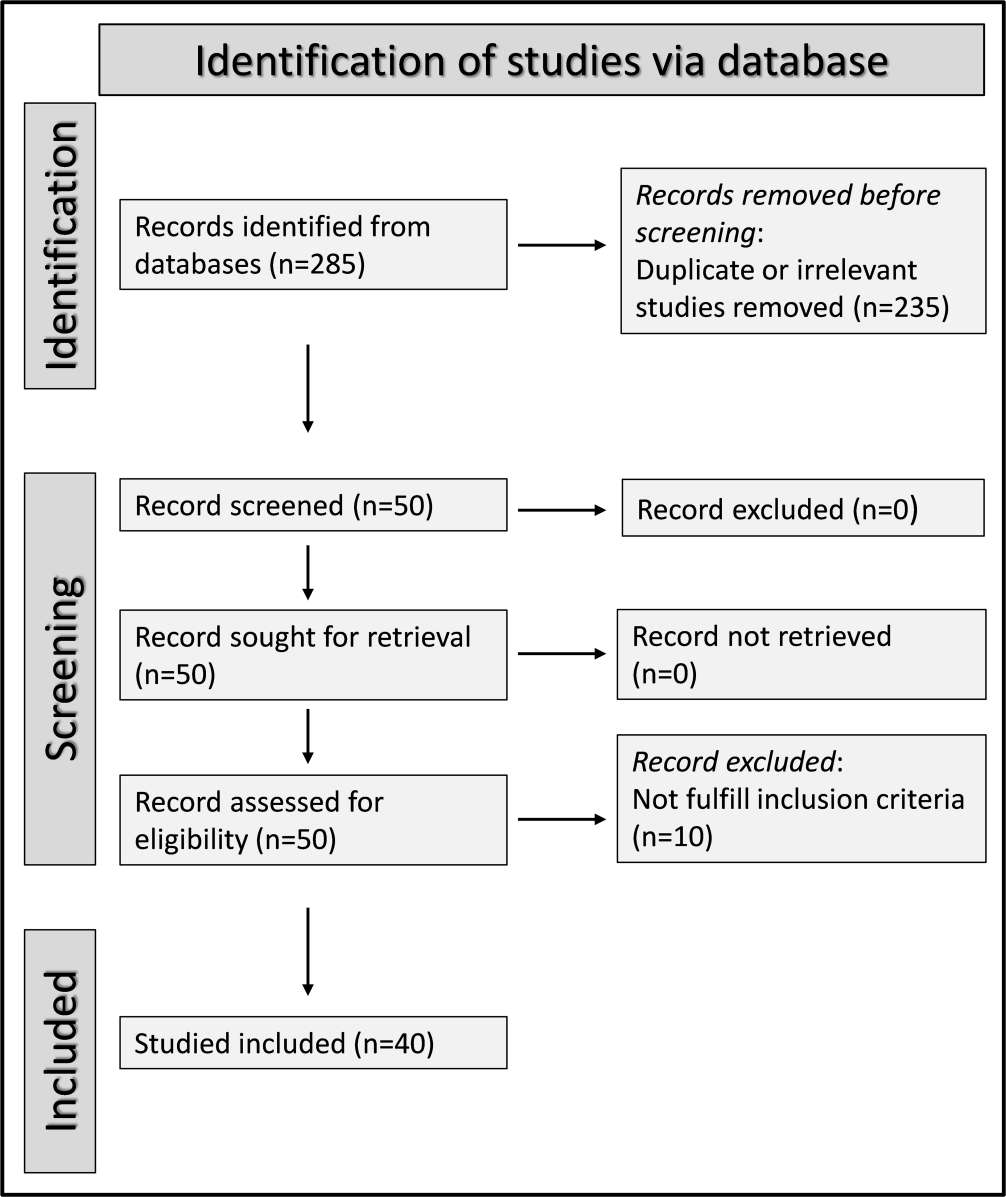
PRISMA 2020 flow chart of study selection.

We initially identified 285 articles. Of them, 235 were excluded because they were either duplicates or irrelevant. Following a full-text review of the remaining 50 articles, a further ten were excluded because they did not meet the inclusion criteria, leaving 40 articles for analysis (**Figure 1**) (21, 36–74). The study design was prospective in three studies (50, 67, 68), unclear in one (66), and retrospective in the remaining 36 (21, 36–49, 51–65, 69–74). The clinical endpoints included mortality in 19 studies (36, 37, 39, 41, 43, 45, 48, 49, 51, 52, 55, 56, 58, 60, 61, 65, 66, 68, 73), and measures of severe disease as follows: disease severity based on existing guidelines in eight (42, 44, 63, 64, 67, 70, 72, 74), transfer to the intensive care unit (ICU) in eight (38, 40, 45, 50, 53, 62, 68, 69), invasive mechanical ventilation in two (46, 52), disease progression in two (57, 71), prolonged hospital stay in one (21), intubation in one (37), deep vein thrombosis in one (54), acute pulmonary embolism in one (54), and acute limb ischemia in one (39). All studies reported the SII on hospital admission.

### Pooled standardized mean differences

#### Study characteristics

Thirty-nine studies (with 45 patient groups) reported the SII in 19,352 patients (mean age 61 years, 55% males) with a non-severe disease/survivor status and 5,524 patients (mean age 69 years, 60% males) with a severe disease/non-survivor status. Twenty-three studies were conducted in Asia (36, 38, 40–42, 48–51, 57, 59, 62–68, 70–74), eleven in Europe (21, 39, 43, 44, 47, 55, 56, 58, 60, 61, 69), four in America (37, 45, 46, 52), and one in Africa (53) (**Table 1**).

**Table 1:** Characteristics of the studies reporting the systemic inflammation index in COVID-19 patients with non-severe disease/survivor status and severe disease/non-survivor status.

Study	Non-severe disease/survivor status				Severe disease/non-survivor status				Endpoint
	n	Age (Years)	M/F	SII (Mean ± SD)	n	Age (Years)	M/F	SII (Mean ± SD)	
Fois AG et al, 2020, Italy (47)	90	68	56/34	1,353 ± 1,295	29	80	21/8	2,375 ± 2,859	Mortality
Luo X et al, 2020, China (59)	214	51	99/115	608 ± 372	84	71	51/33	1,271 ± 818	Mortality
Rokni M et al, 2020, Iran (66)	205	NR	129/76	1,164 ± 1,473	28	NR	20/8	3,533 ± 2,991	Mortality
Xue G et al, 2020, China (72)	56	60.5	30/26	705 ± 555	58	64	34/24	1,370 ± 994	Severity
Zhao Y et al, 2020, China (74)	211	64	96/115	717 ± 606	74	68	38/36	1,663 ± 2,052	Severity
Acar E et al, 2020, Turkey (36)	129	58	42/87	2,445 ± 8,575	19	70	14/5	4,426 ± 2,746	Mortality
Gujar RK et al, 2021, India (50)	577	46	381/196	653 ± 750	264	59	171/93	2,084 ± 2,527	ICU
Li Y et al, 2021, China (57)	409	61	208/201	931 ± 734	56	67	40/16	2,130 ± 2,024	Progression
López-Escobar A et al, 2021, Spain (58)	1,767	66	1,032/735	960 ± 800	321	83	213/108	1,840 ± 1,780	Mortality
Moisa E et al, 2021, Romania (60)	130	66.8	93/37	3,440 ± 2,868	142	58.2	93/49	4,169 ± 3,073	Mortality
Nalbant A et al, 2021, Turkey (62)	78	58	37/41	590 ± 391	40	70	23/17	1,269 ± 990	ICU
San I et al, 2021, Turkey (67)	344	68	192/152	485 ± 334	44	42	27/17	1,161 ± 1,356	Severity
Sevinc C et al. (a), 2021, Turkey (68)	86	60	41/45	1,078 ± 1,196	31	65	16/15	2,164 ± 1,613	ICU
Sevinc C et al. (b), 2021, Turkey (68)	88	59	41/47	1,130 ± 1,200	29	67	16/13	2,138 ± 1,707	Mortality
Velazquez S et al, 2021, Spain (69)	2069	70	1,202/867	1,047 ± 930	185	68	138/47	1,567 ± 1,422	ICU
Xu J et al, 2021, China (71)	260	56	150/110	496 ± 379	78	62.5	42/36	899 ± 792	Progression
Zinellu A et al, 2021, Italy (21)	43	66	27/16	1,068 ± 966	22	69	16/6	1,653 ± 1,374	LOS
Alagbe AE et al. (a), 2022, Brazil (37)	257	56	152/105	2,300 ± 444	63	65	39/24	3,267 ± 1,481	Mortality
Alagbe AE et al. (b), 2022, Brazil (37)	202	56	NR	1,930 ± 520	118	60	NR	3,430 ± 1,037	Intubation
Alkhatib B et al, 2022, Jordan (38)	94	47	55/39	1,788 ± 1,422	14	53	9/5	2,436 ± 1,174	ICU
Arbanasi EM et al. (a), 2022, Romania (39)	461	70	284/177	610 ± 572	49	74	21/28	3,026 ± 2,632	ALI
Arbanasi EM et al. (b), 2022, Romania (39)	396	70	247/149	831 ± 598	114	73	58/56	2,515 ± 1,722	Mortality
Asaduzzaman M et al, 2022, Bangladesh (40)	344	59	231/113	1,001 ± 838	98	65	60/38	1,948 ± 2,041	ICU
Çelikkol A et al, 2022, Turkey (42)	31	NR	NR	537 ± 392	25	NR	NR	683 ± 670	Severity
Citu C et al, 2022, Romania (43)	91	62	NR	2,183 ± 1,847	17	70	NR	2,798 ± 2,429	Mortality
Cocos R et al, 2022, Romania (44)	183	51	97/86	1,038 ± 1,314	71	67	44/27	2,819 ± 2,448	Severity
Farias JP et al. (a), 2022, Brazil (45)	1,060	52	477/583	988 ± 1,235	476	68	260/216	2,947 ± 7,867	ICU
Farias JP et al. (b), 2022, Brazil (45)	1,342	54	630/712	1,293 ± 2,070	193	74	107/86	3,698 ± 6,209	Mortality
Ghobadi H et al. (a), 2022, Iran (48)	947	48	548/399	932 ± 788	135	54	88/47	1,856 ± 1,607	Mortality
Ghobadi H et al. (b), 2022, Iran (48)	492	76	238/254	1,077 ± 871	218	78	114/104	1,136 ± 1,400	Mortality
Gozdas HT et al, 2022, Turkey (49)	86	66	49/37	3,145 ± 2,670	262	76	156/106	3,780 ± 4,001	Mortality
Gunay S et al, 2022, Turkey (51)	220	56	111/109	1,165 ± 981	45	70	25/20	4,050 ± 3,468	Mortality
Gutiérrez-Pérez IA et al. (a), 2022, Mexico (52)	352	NR	NR	3,326 ± 2,353	196	NR	NR	4,792 ± 3,269	IMV
Gutiérrez-Pérez IA et al. (b), 2022, Mexico (52)	491	NR	NR	4,263 ± 2,987	316	NR	NR	5,271 ± 3,627	Mortality
Hamad DA et al, 2022, Egypt (53)	185	33	91/94	492 ± 804	310	58	181/129	2,016 ± 2,163	ICU
Kudlinski B et al, 2022, Poland (56)	177	57	114/63	3,666 ± 3,381	108	63	75/33	4,554 ± 3,512	Mortality
Muresan AV et al, 2022, Romania (61)	746	70	397/349	1,369 ± 1,190	143	72	77/66	5,010 ± 4,273	Mortality
Poorhaji MM et al, 2022, Iran (63)	42	53	26/16	991 ± 1,024	67	58	47/20	1,704 ± 1,379	Severity
Prasad S et al, 2022, India (64)	948	53	NR	1,134 ± 1,573	285	79	NR	3,329 ± 3,817	Severity
Qiu W et al, 2022, China (65)	2,290	72	1,331/959	469 ± 443	57	84	38/19	1,236 ± 1,040	Mortality
Xia W et al, 2022, China (70)	77	45	43/34	566 ± 386	48	56	28/20	2,990 ± 3,199	Severity
Cakirka G et al, 2023, Turkey (41)	733	47	346/387	600 ± 463	94	72	62/32	2,003 ± 1,994	Mortality
Fernandes NF et al, 2023, Brazil (46)	83	61	50/33	1,749 ± 1,416	129	61	81/48	3,438 ± 3,215	IMV
Khadzhieva MB et al, 2023, Russia (55)	138	57	73/55	835 ± 923	31	62	18/13	1,503 ± 1,959	Mortality
Yilmaz A et al, 2023, Turkey (73)	128	70	65/63	2,951 ± 4,037	338	73	200/138	3,583 ± 4,423	Mortality

ALI, acute limb ischemia; F, female; ICU, admission to the intensive care unit; IMV, invasive mechanical ventilation; LOS, length of stay; M, male; NR, not reported; SII, systemic inflammation index.

#### Risk of bias

The risk of bias was low in 29 studies (21, 36, 37, 39–41, 43–45, 47, 49, 51–53, 55–61, 64, 65, 67, 69, 71–74) and high in the remaining ten (38, 42, 46, 48, 50, 62, 63, 66, 68, 70) (**Supplementary Table 3**).

#### Results of individual studies and syntheses

**Figure 2:**
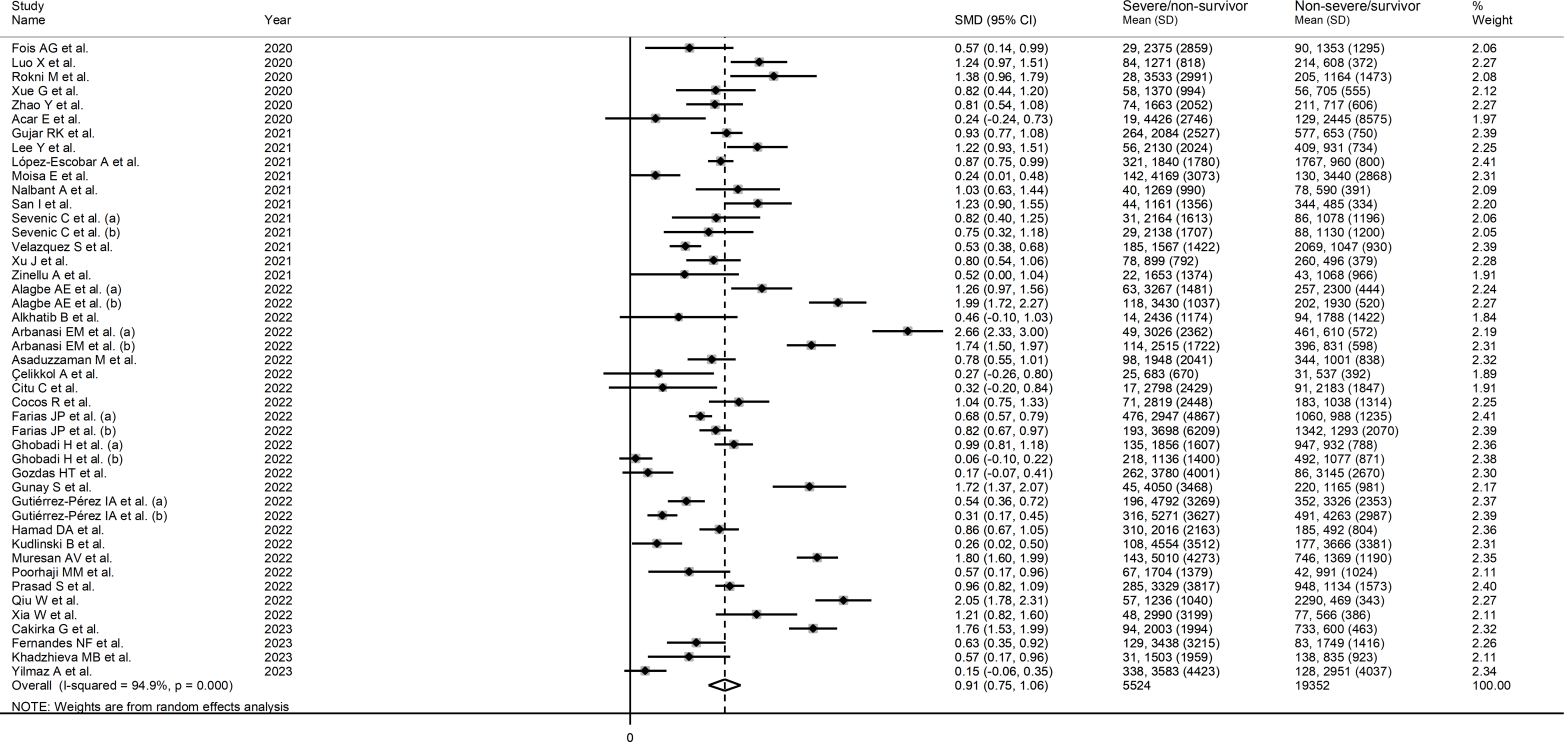
Forest plot of studies reporting SII values in COVID-19 patients with different disease severity and survival status.

The forest plot of SII values in patients with a non-severe disease/survivor status and patients with a severe disease/non-survivor status is shown in **Figure 2**. Random-effects models were used because of the extreme heterogeneity observed (I^2 ^= 94.9%, p<0.001). Pooled results showed that patients with a severe disease/non-survivor status had significantly higher SII values than patients with a non-severe disease/survivor status (SMD=0.91, 95% CI 0.75 to 1.06, p<0.001). In sensitivity analysis, the corresponding pooled SMD values were not substantially altered when individual studies were sequentially omitted (effect size ranged between 0.87 and 0.93, **Supplementary Figure 1**).

#### Publication bias

There was no publication bias according to the Begg’s (p=0.87) and the Egger’s (p=0.18) test. However, the “trim-and-fill” method identified one missing study to be added to the left side of the funnel plot to ensure symmetry (**Supplementary Figure 2**). The resulting effect size, however, was similar to the primary analysis (SMD=0.87, 95% CI 0.71 to 1.03, p<0.001).

#### Sub-group and meta-regression analysis

In meta-regression, significant correlations were observed between the SMD and albumin (t=-3.96, p=0.002), lactate dehydrogenase (t=2.16, p=0.048), creatinine (t=2.53 p=0.02), and D-dimer (t=2.62, p=0.017). By contrast, no significant correlations were observed with age (t=​0.66, p​=0.51), sex (t​=​-1.26, p=0.21), publication year (t=0.35, p​= 0.73), study design (t​=0.04, p=​0.97), sample size (t=1.11, 0.27), C-reactive protein (t=1.73, p​=0.10), ferritin (t=0.87, p=0.41), and history of diabetes (t​=​-0.16, p=0.88), hypertension (t​=0.12, p​=0.90), or cardiovascular disease (t​=-0.33, p=0.74).

In sub-group analysis, there were no significant differences in the pooled SMD between studies reporting disease severity (SMD=0.91, 95% CI 0.74 to 1.07, p<0.001; I^2 ^= 96.6%, p<0.001), survival status (SMD=0.88, 95% CI 0.61 to 1.14, p<0.001; I^2 ^= 56.5%, p=0.023), and ICU admission (SMD= 0.76, 95% CI 0.64 to 0.89, p<0.001; I^2 ^= 63.3%, p=0.008; **Supplementary Figure 3**). However, the between-study variance was relatively lower in studies reporting severity and ICU admission (I^2 ^= 56.5% and 63.3%, respectively). Similarly, there were no significant differences in the pooled SMD between studies performed in Europe (SMD=0.94, 95% CI 0.56 to 1.31, p<0.001; I^2 ^= 96.6%, p<0.001), Asia (SMD=0.89, 95% CI 0.69 to 1.10, p<0.001; I^2 ^= 93.5%, p<0.001) and America (SMD=0.92, 95% CI 0.56 to 1.28, p<0.001; I^2 ^= 96.3%, p<0.001; **Supplementary Figure 4**).

#### Certainty of evidence

The initial level of certainty was considered low because of the cross-sectional nature of the studies (rating 2, ⊕⊕⊝⊝). After taking into account the low risk of bias in the majority of studies (no rating change), the substantial but partly explainable heterogeneity (no rating change), the lack of indirectness (no rating change), the relatively low imprecision (confidence intervals not crossing the threshold, no rating change), the relatively large effect size (SMD=0.91, upgrade one level), and the absence of publication bias (no rating change), the overall level of certainty was upgraded to moderate (rating 3, ⊕⊕⊕⊝).

### Pooled odds ratios

#### Study characteristics

Ten studies (13 patient groups) in 9,851 COVID-19 patients (56% males, mean age 67 years), all retrospective, reported associations between the SII and disease severity or survival status expressed as ORs in multivariate logistic regression analysis (36, 44, 52–54, 58, 61, 69, 72, 74). The study endpoint was mortality in five studies (36, 52, 54, 58, 61), disease severity based on existing clinical guidelines in three (44, 72, 74), transfer to ICU in two (53, 69), invasive mechanical ventilation in one (52), deep vein thrombosis in one (61), and acute pulmonary embolism in one (61). Four studies were conducted in Europe (44, 58, 61, 69), four in Asia (36, 54, 72, 74), one in America (52), and one in Africa (53) (**Table 2**).

**Table 2:** Studies investigating the association between the systemic inflammation index and disease severity or mortality in COVID-19 patients using odds ratios.

Study	n	Age (Years)	M/F	Odds ratio	95% CI	Endpoint
Xue G et al, 2020, China (72)	114	62	64/50	1.003	1.002-1.004	Severity
Zhao Y et al, 2020, China (74)	285	66	172/187	7.04	2.57-19.28	Severity
Acar E et al, 2020, Turkey (36)	148	59	56/92	10.651	3.828-29.634	Mortality
López-Escobar A et al, 2021, Spain (58)	2,088	69	1,245/843	1.02	1.01-1.03	Mortality
Velazquez S et al, 2021, Spain (69)	2,254	69	1,340/914	1.01	0.995-1.02	ICU
Karaaslan et al, 2022, Turkey (54)	191	54	94/97	1.001	1.000-1.004	Mortality
Cocos R et al, 2022, Romania (44)	254	56	141/113	1	1.000-1.001	Severity
Gutiérrez-Pérez IA et al. (a), 2022, Mexico (52)	548	NR	NR	1.29	0.91-1.83	IMV
Gutiérrez-Pérez IA et al. (b), 2022, Mexico (52)	807	59	NR	1.5	1.1.-2.06	Mortality
Hamad DA et al, 2022, Egypt (53)	495	49	272/223	1.008	0.626-1.967	ICU
Muresan AV et al. (a), 2022, Romania (61)	889	71	474/415	11.42	7.36-17.72	Mortality
Muresan AV et al. (b), 2022, Romania (61)	889	71	474/415	9.33	6.35-13.71	DVT
Muresan AV et al. (c), 2022, Romania (61)	889	71	474/415	5.09	2.8-9.26	APE

APE, acute pulmonary embolism; CI, confidence interval; DVT, deep vein thrombosis; F, female; ICU, admission to the intensive care unit; IMV, invasive mechanic ventilation; M, male; NR, not reported.

#### Risk of bias

The risk of bias was low in all studies (36, 44, 52–54, 58, 61, 69, 72, 74) (**Supplementary Table 3**).

#### Results of individual studies and syntheses

**Figure 3:**
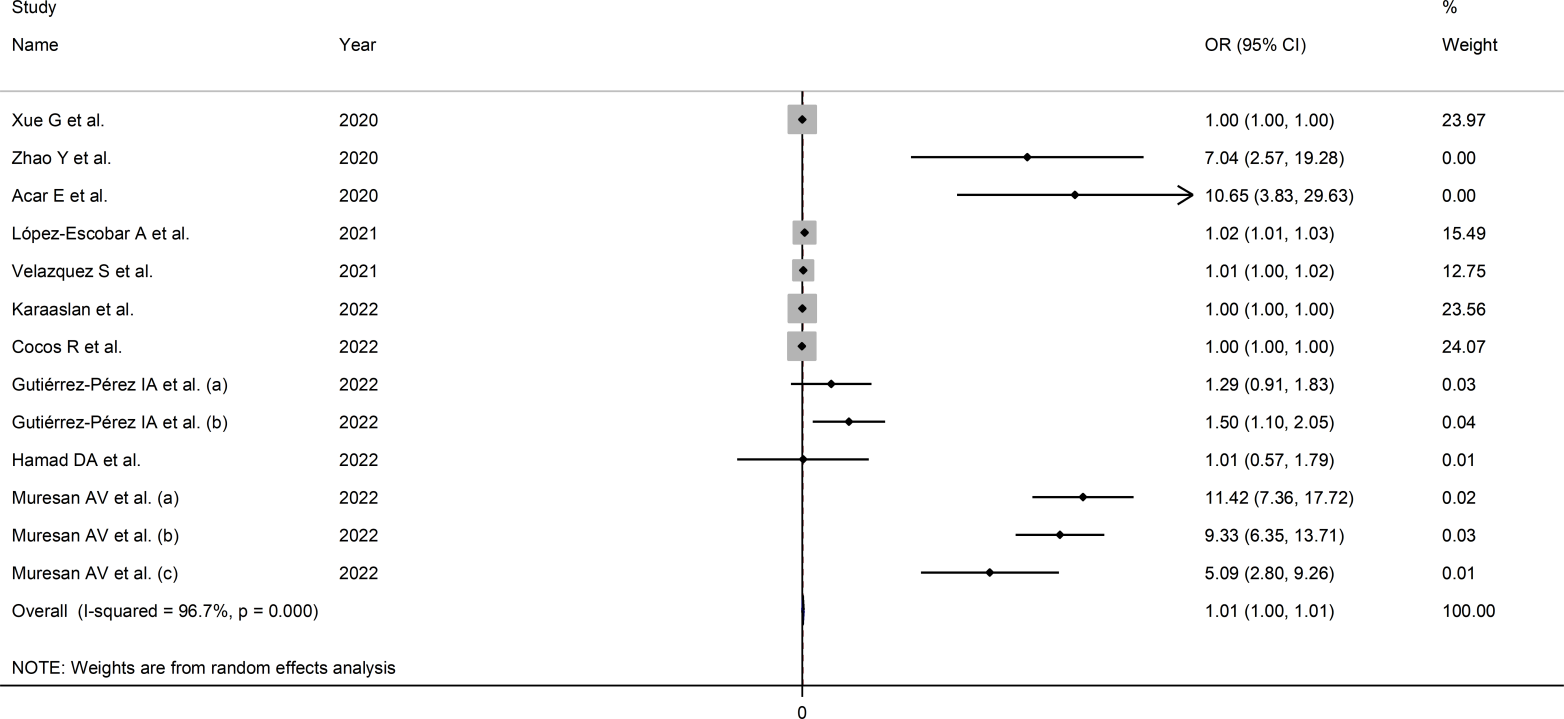
Forest plot of studies examining the association between the SII and disease severity or survival status in patients with COVID-19 by means of odds ratio.

The extreme between-study heterogeneity observed (I^2 ^= 96.7%, p<0.001) required the use of random-effects models. Pooled results showed that a higher SII was significantly associated with severe disease or mortality (OR=1.007, 95% CI 1.001 to 1.014, p=0.032; **Figure 3**). In sensitivity analysis, the corresponding pooled ORs were influenced by two studies (44, 72) (**Supplementary Figure 5**). Their removal was associated with a slight increase in the effect size (OR=1.14, 95% CI 1.08-1.20, p=0.001; I^2 ^= 97%, p<0.001; **Supplementary Figure 6**).

#### Publication bias

There was evidence of publication bias according to the Egger’s (p=0.004) but not the Begg’s (p=0.213) test.

#### Sub-group and meta-regression analysis

In meta-regression analysis, there were no significant correlations between the OR and age (t=0.54, p=0.60), sex (t=0.17, p=0.87), publication year (t=-0.34, p=0.74), or sample size (t=0.61, p=0.56). Meta-regression of other parameters could not be conducted because of the limited information available.

In sub-group analysis, the SII was significantly associated with mortality (OR=1.08, 95% CI 1.02 to 1.15, p=0.013; I^2 ^= 96.6%, p<0.001) but not measures of disease severity (OR=1.00, 95% CI 1.00 to 1.01, p=0.243; I^2 ^= 97.5%, p<0.001; **Supplementary Figure 7**). Furthermore, the effect size was significant in European (OR=1.10, 95% CI 1.04 to 1.16, p<0.001; I^2 ^= 98.3%, p<0.001) and American (OR=1.40, 95% CI 1.11 to 1.77, p=0.005; I^2 ^= 0.0%, p=0.529), but not in Asian studies (OR=1.00, 95% CI 1.00 to 1.01, p=0.52; I^2 ^= 92.1%, p<0.001; **Supplementary Figure 8**). Notably, the heterogeneity was virtually absent in American studies (I^2 ^= 0.0%).

#### Certainty of evidence

The initial level of certainty was considered low because of the cross-sectional nature of the studies (rating 2, ⊕⊕⊝⊝). After taking into account the low risk of bias in all studies (no rating change), the substantial but partly explainable heterogeneity (no rating change), the lack of indirectness (no rating change), the relatively low imprecision (confidence intervals not crossing the threshold, no rating change), the relatively small effect size (OR=1.007, no rating change), and the presence of publication bias (downgrade by one level), the overall level of certainty was downgraded to very low (rating 1, ⊕⊝⊝⊝).

### Pooled hazard ratios

#### Study characteristics

Six studies (with seven patient groups) in 5,100 COVID-19 patients (58% males, mean age 66 years), all retrospective, reported associations between the SII and disease severity or survival status as HRs using multivariate logistic regression analysis (47, 48, 56, 60, 65, 74). The studied endpoint was mortality in five studies (47, 48, 56, 60, 65) and severity in the remaining one (74). Three studies were conducted in Asia (48, 65, 74) and three in Europe (47, 56, 60) (**Table 3**).

**Table 3:** Studies investigating the association between the systemic inflammation index and disease severity or mortality in COVID-19 patients using hazard ratio.

Study	n	Age (Years)	M/F	Hazard ratio (95% CI)	Endpoint
Fois AG et al, 2020, Italy (47)	119	72	77/42	1.0001 (1.0000-1.0001)	Mortality
Zhao Y et al, 2020, China (74)	285	66	134/151	2.00 (0.90-4.42)	Severity
Moisa E et al, 2021, Romania (60)	272	62	186/116	1.68 (1.13-2.49)	Mortality
Ghobadi H et al. (a), 2022, Iran (48)	1,082	48	636/446	4.90 (3.401-7.060)	Mortality
Ghobadi H et al. (b), 2022, Iran (48)	710	76	352/358	2.823 (2.132-3.739)	Mortality
Kudlinski B et al, 2022, Poland (56)	285	59	189/96	0.606 (0.390-0.943)	Mortality
Qiu W et al, 2022, China (65)	2,347	72	1,369/978	4.591 (2.595-8.120)	Mortality

CI, confidence interval; F, female; M, male.

#### Risk of bias

The risk of bias was low in all studies, barring one (48) (**Supplementary Table 3**).

#### Results of individual studies and syntheses

**Figure 4:**
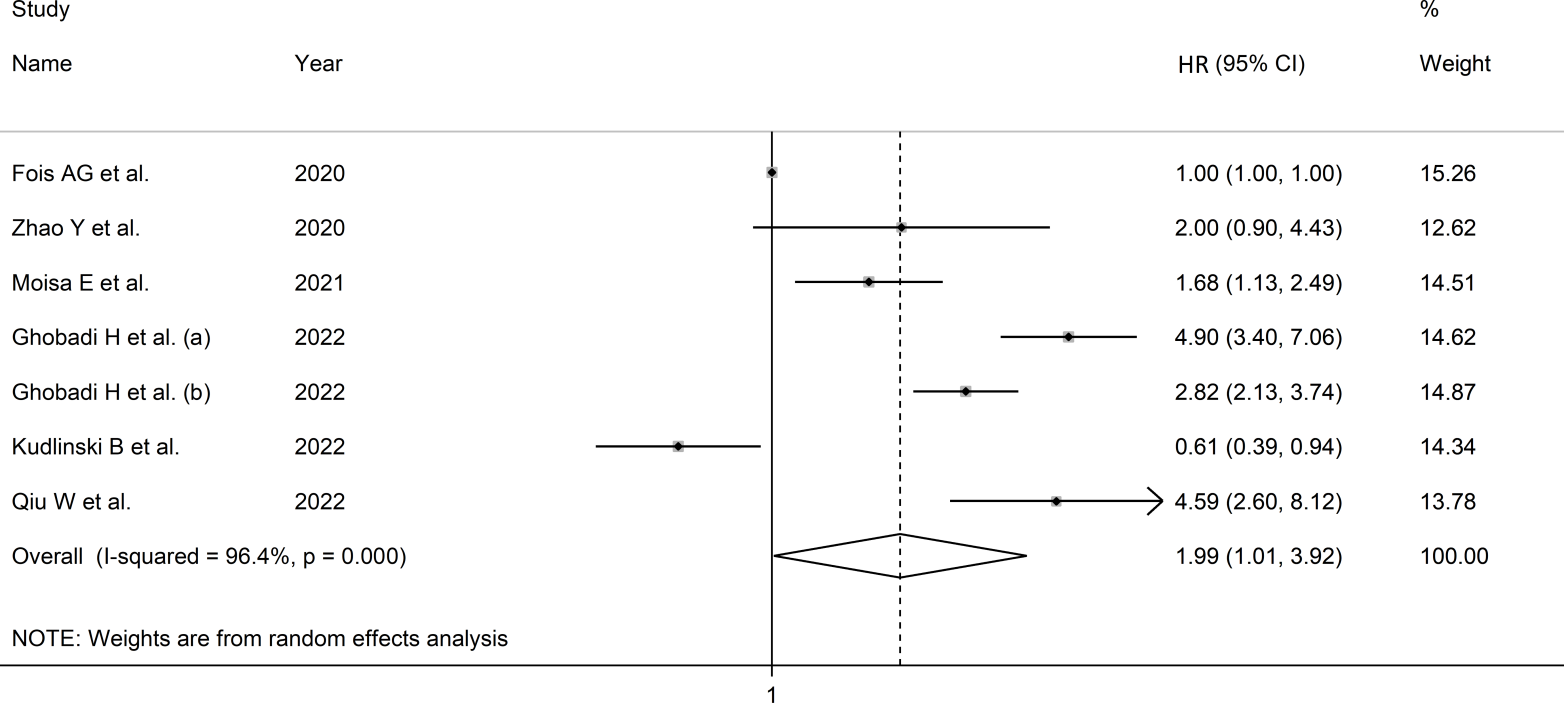
Forest plot of studies examining association between SII and disease severity or survival status in patients with COVID-19 through HR.

Due to the extreme heterogeneity between studies (I^2 ^= 96.4%, p<0.001), random-effects models were used. Pooled results showed that a higher SII was significantly associated with severe disease and mortality (HR=1.99, 95% CI 1.01 to 3.92, p=0.047; **Figure 4**). In sensitivity analysis, the corresponding pooled HRs were not substantially altered when individual studies were removed, suggesting that the results of the meta-analysis were stable (HR ranged between 1.70 and 2.43) (**Supplementary Figure 9**).

#### Publication bias

Assessment of publication bias was not possible because of the relatively small number of studies.

#### Sub-group and meta-regression analysis

Meta-regression analysis was not possible because of the relatively small number of studies. In sub-group analysis, the effect size was significant in Asian (HR=3.52, 95% CI 2.41 to 5.12, p<0.001; I^2 ^= 63.7%, p=0.043), but not European studies (HR=1.01, 95% CI 0.39 to 2.59, p=0.986; I^2 ^= 82.7%, p<0.001; **Supplementary Figure 10**), with a lower between-studies variance in the former.

#### Certainty of evidence

The initial level of certainty was considered low because of the cross-sectional nature of the studies (rating 2, ⊕⊕⊝⊝). After taking into account the low risk of bias in the majority of studies (no rating change), the substantial but partly explainable heterogeneity (no rating change), the lack of indirectness (no rating change), the relatively low imprecision (confidence intervals not crossing the threshold, no rating change), the moderate effect size (HR=1.99, no rating change), and the presence of publication bias (downgrade by one level), the overall level of certainty was downgraded to very low (rating 1, ⊕⊝⊝⊝).

### Accuracy of the SII in predicting severe disease or mortality

#### Study characteristics

Twenty-one studies (28 patient groups) in 17,863 COVID-19 patients (60% males, mean age 66 years), all retrospective, reported sensitivity and specificity of the ability of the SII to predict severe disease or mortality (36–40, 42, 45, 47, 48, 51, 53, 54, 56, 58, 61, 64, 68–70, 72, 74). Twelve studies were performed in Asia (36, 38, 40, 42, 48, 51, 54, 64, 68, 70, 72, 74), six in Europe (39, 47, 56, 58, 61, 69), two in America (37, 45), and one in Africa (53). The studied endpoint was mortality in 12 studies (36, 37, 39, 45, 47, 48, 51, 54, 56, 58, 61, 68), ICU admission in seven (38–40, 45, 53, 68, 69), disease severity based on existing guidelines in five (42, 64, 70, 72, 74), acute pulmonary embolism in one (61), acute limb ischemia in one (39), and deep vein thrombosis in one (61) (**Table 4**).

**Table 4:** Studies investigating the accuracy of the systemic inflammation index in predicting severe disease and mortality in COVID-19 patients.

Study	n	AUC (95% CI)	Cut-off	Sensitivity (%)	Specificity (%)	Endpoint
Fois AG et al, 2020, Italy (47)	119	0.628 (0.534-0.715)	1,835	0.55	0.75	Mortality
Xue G et al, 2020, China (72)	114	0.72 (NR)	809	0.724	0.679	Severity
Zhao Y et al, 2020, China (74)	285	0.72 (NR)	1,091	0.77	0.61	Severity
Acar E et al, 2020, Turkey (36)	148	0.742 (0.600-0.864)	2,699	0.684	0.775	Mortality
López-Escobar A et al, 2021, Spain (58)	2,088	NR	1,387	0.57	0.71	Mortality
Sevinc C et al. (a), 2021, Turkey (68)	117	0.752 (0.644-0.861)	1,145	0.68	0.67	ICU
Sevinc C et al. (b), 2021, Turkey (68)	117	0.714 (0.596-0.832)	1,145	0.64	0.64	Mortality
Velazquez S et al, 2021, Spain (69)	2,254	0.599 (0.506-0.595)	1,226	0.55	0.65	ICU
Karaaslan et al, 2022, Turkey (54)	191	0.751 (NR)	618.8	0.8	0.615	Mortality
Alagbe AE et al, 2022, Brazil (37)	320	0.61 (0.54-0.69)	1,450	0.65	0.55	Mortality
Alkhatib B et al, 2022, Jordan (38)	320	0.645 (0.547-0.735)	1,695	0.714	0.591	ICU
Arbanasi EM et al. (a), 2022, Romania (39)	510	0.888 (0.834-0.942)	2,219	0.816	0.872	ALI
Arbanasi EM et al. (b), 2022, Romania (39)	510	0.850 (0.811-0.889)	1,347	0.825	0.778	Mortality
Arbanasi EM et al. (c), 2022, Romania (39)	510	0.779 (0.736-0.521)	1,413	0.663	0.851	ICU
Asaduzzaman M et al, 2022, Bangladesh (40)	442	0.651 (NR)	1,981	0.49	0.78	ICU
Çelikkol A et al, 2022, Turkey (42)	56	0.555 (NR)	425	0.65	0.348	Severity
Farias JP et al. (a), 2022, Brazil (45)	1,361	0.77 (0.74-0.79)	728	0.8	0.6	ICU
Farias JP et al. (b), 2022, Brazil (45)	1,539	0.75 (0.70-0.79)	735	0.84	0.53	Mortality
Ghobadi H et al. (a), 2022, Iran (48)	1,082	0.848 (0.825-0.869)	1,994	0.741	0.872	Mortality
Ghobadi H et al. (b), 2022, Iran (48)	710	0.800 (0.769-0.829)	1,868	0.702	0.804	Mortality
Gunay S et al, 2022, Turkey (51)	265	0.794 (NR)	1,134	0.667	0.79	Mortality
Hamad DA et al, 2022, Egypt (53)	495	0.819 (0.782-0.856)	1,346	0.509	0.956	ICU
Kudlinski B et al, 2022, Poland (56)	285	0.576 (NR)	2,058	0.731	0.452	Mortality
Muresan AV et al. (a), 2022, Romania (61)	889	0.836 (0.800-0.871)	2,209	0.797	0.744	Mortality
Muresan AV et al. (b), 2022, Romania (61)	889	0.805 (0.768-0.842)	1,890	0.791	0.842	DVT
Muresan AV et al. (c), 2022, Romania (61)	889	0.761 (0.694-0.828)	1,840	0.758	0.619	APE
Prasad S et al, 2022, India (64)	1,233	0.793 (NR)	999	0.772	0.292	Severity
Xia W et al, 2022, China (70)	125	0.86 (0.790-0.931)	887	0.8125	0.8182	Severity

ALI, acute limb ischemia; APE, acute pulmonary embolism; AUC, area under the curve; CI, confidence interval; DVT, deep vein thrombosis; ICU, transfer to the intensive care unit; NR, not reported.

#### Risk of bias

The risk of bias was low in all studies, barring five (38, 42, 48, 68, 70) (**Supplementary Table 3**).

#### Results of individual studies and syntheses

**Figure 5:**
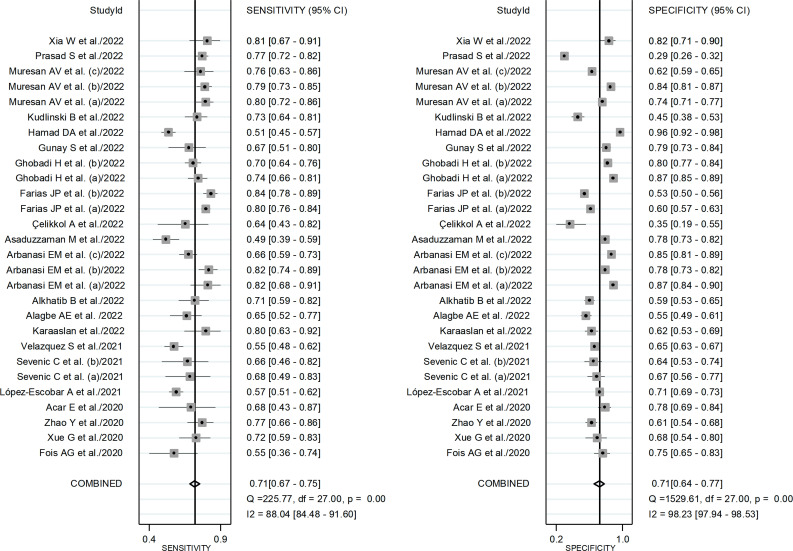
Forest plot for the pooled estimates of sensitivity and specificity of the SII towards disease severity or mortality.

**Figure 6:**
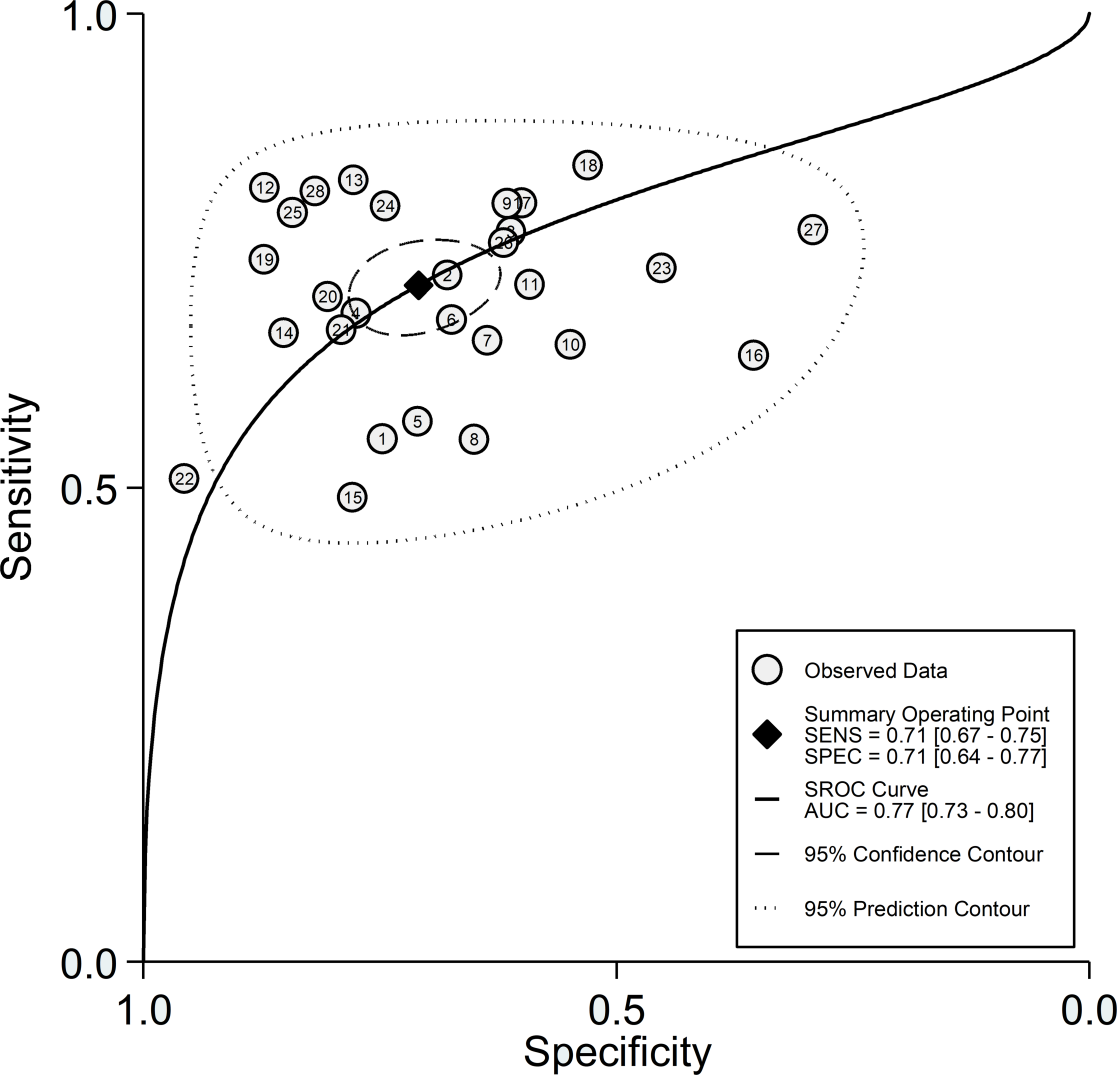
SROC curve with 95% confidence region and prediction region for the SII towards prediction of severe disease or mortality.

**Figure 7:**
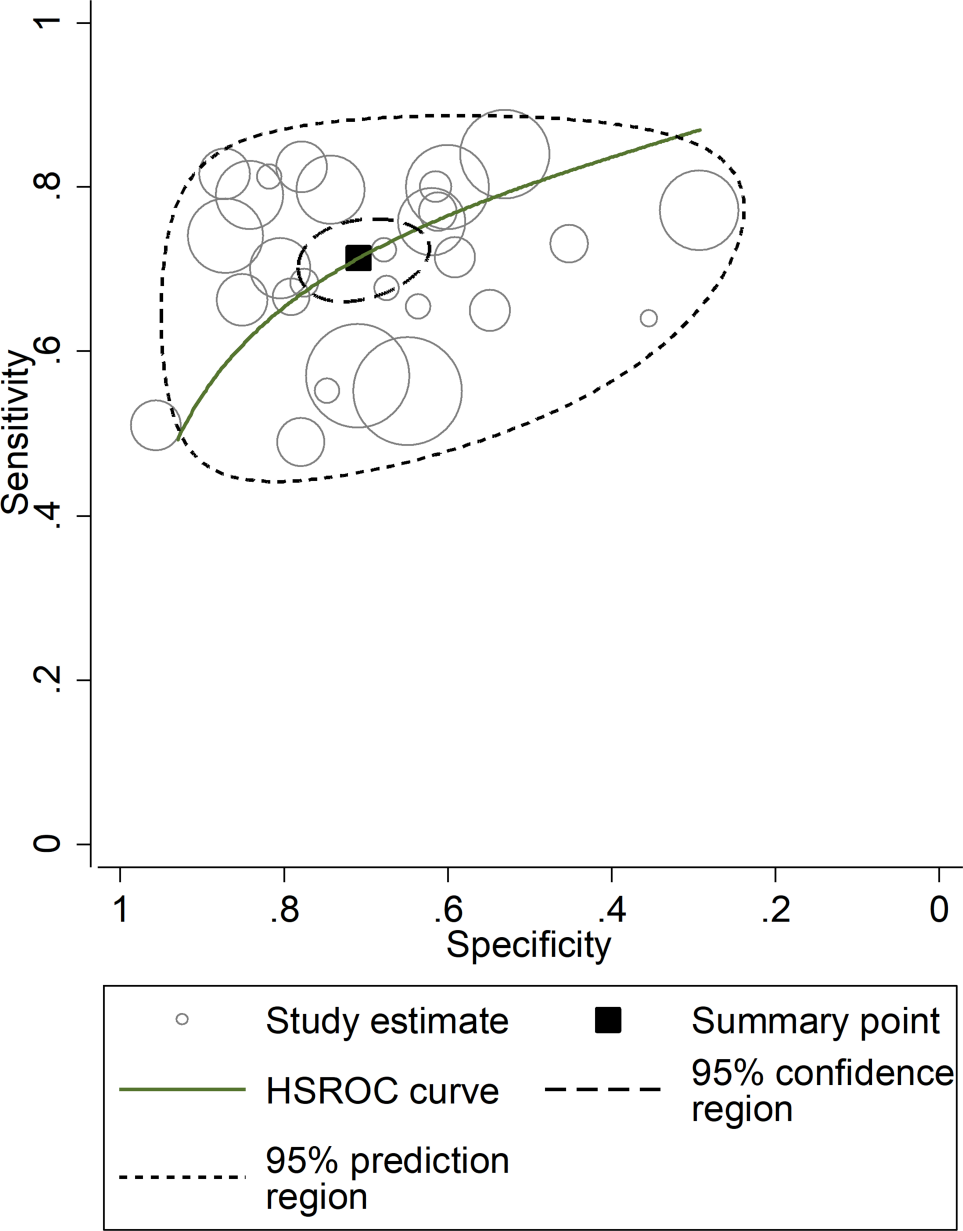
Empirical Bayes (posterior prediction) estimates hierarchical receiving operating characteristic (HSROC) curve with 95% confidence region and prediction region for SII towards prediction of severe disease or mortality.

**Figure 8:**
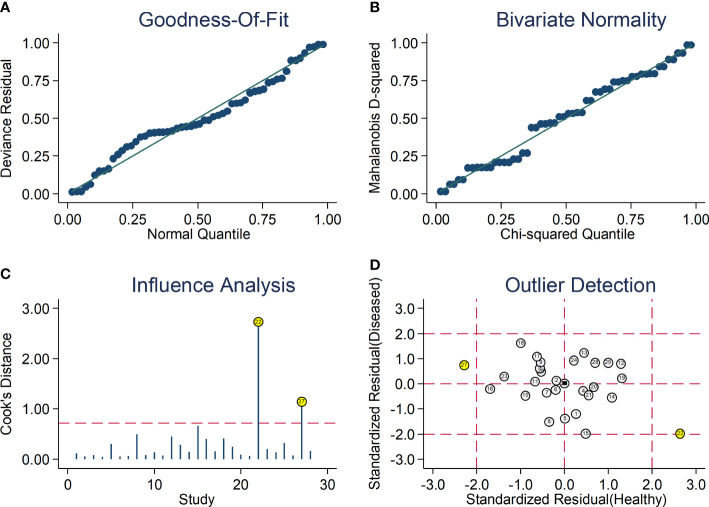
Graphical illustration of residual-based goodness-of-fit **(A)**, bivariate normality **(B)**, influence **(C)**, and outlier detection **(D)** analyses.

After creating forest plots for pooled sensitivity and specificity, a summary receiver operating characteristic (SROC) curve was generated using the HSROC model (midas or metandi command). The pooled sensitivity and specificity for the SII towards severe disease or mortality was 0.71 (95% CI 0.67 to 0.75) and 0.71 (95% CI 0.64 to 0.77), respectively (**Figure 5**). The SROC curve with 95% confidence region and prediction region is described in **Figure 6**. The AUC value was 0.77 (95% CI 0.73 to 0.80), with the summary operating point at a sensitivity of 0.71 and a specificity of 0.71. We also generated empirical Bayes estimates in HSROC analysis, which provide the best estimates of the true sensitivity and specificity in each study (**Figure 7**). The midas command was used to evaluate the quantile plot of residual based goodness-of fit, the Chi-squared probability plot of squared Mahalanobis distances for the assessment of the bivariate normality assumption, the spikeplot for assessing particularly influential observations using Cook’s distance, and a scatter plot to check for outliers using standardized predicted random effects (**Figure 8**). The analysis identified two outliers (53, 64). However, their removal did not exert tangible effects on the results, with AUC of 0.77, sensitivity of 0.72, and specificity of 0.71.

**Figure 9:**
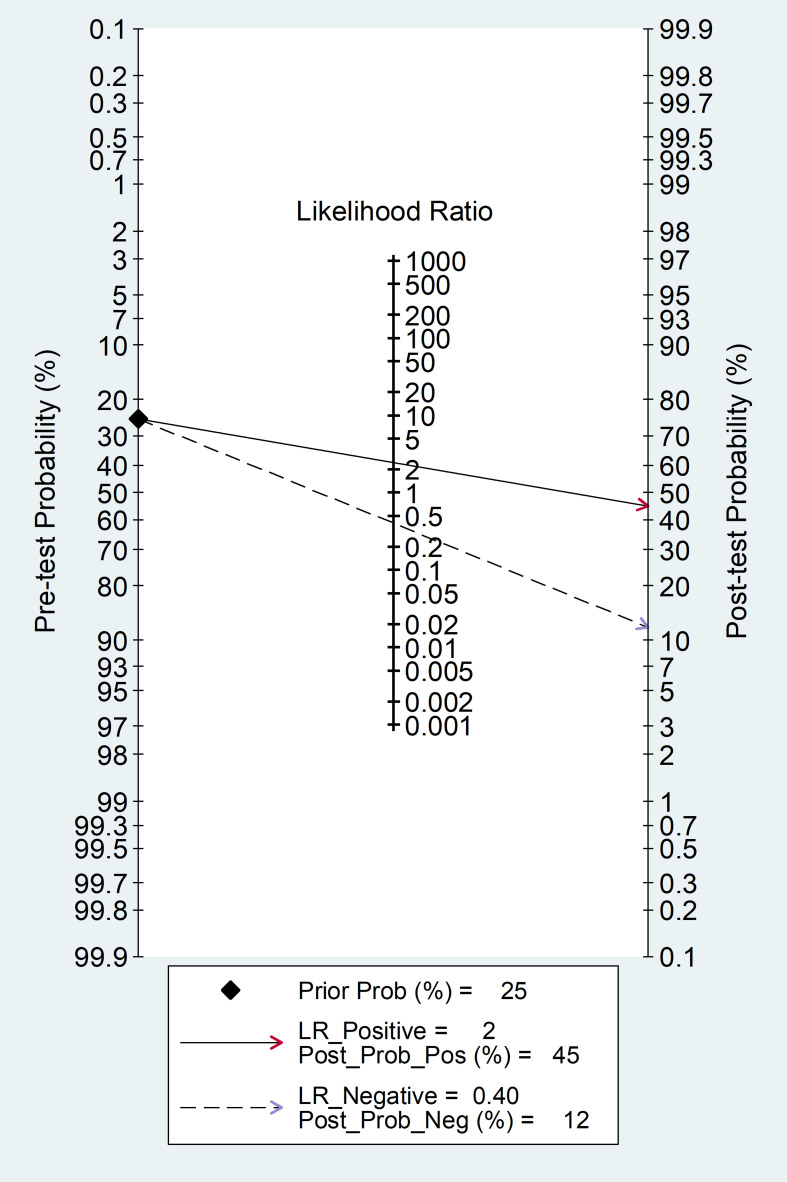
Fagan’s nomogram for the SII towards prediction of severe disease or mortality.

The clinical utility of the SII at the population level was assessed by generating the Fagan’s nomogram (**Figure 9**). Assuming a 25% prevalence of adverse outcomes (pre-test probability), the nomogram showed that the posterior (post-test) probability of adverse outcome was 45% in patients with a relatively high SII and 12% in patients with a relatively low SII.

#### Publication bias

No significant publication bias (p=0.687) was observed in the Deeks’ funnel plot asymmetry test (**Supplementary Figure 11**).

#### Heterogeneity, sub-group, and meta-regression analysis

The presence of heterogeneity across the studies was investigated using different approaches. First, the HSROC curve was shown to be symmetric, based on the correlation coefficient between logit transformed sensitivity and specificity (HSROC model), which was negative (-0.277, 95% CI -0.616 to -0.149). However, the symmetry parameter β (0.587, 95% CI 0.169 to 1.00) showed a significant p-value (0.006), suggesting an elevated between-study variance (data not reported). In addition, the visual representation of the HSROC suggested a moderate degree of heterogeneity (95% CI 0.73 to 0.80; **Figure 7**). In midas, the pooled sensitivity and specificity showed an inconsistency (I^2^) of 88.04% and 98.23%, respectively (**Figure 5**). Using the bivariate boxplot with logit_Se and logit_Sp, four studies (42, 45, 53, 64) fell outside the circles, which also indicates heterogeneity (**Supplementary Figure 12**). The sources of heterogeneity were further explored using univariate meta-regression analysis. Age and sex were significantly associated with the effect size for sensitivity (p=0.02 and p=0.01, respectively) whereas no parameter was significantly associated with the effect size for specificity (**Supplementary Figure 13**).

## Discussion

Our systematic review and meta-analysis has shown that the SII on admission was significantly higher in hospitalized patients with COVID-19 with a severe disease or non-survivor status when compared to patients with a non-severe disease or survivor status. Notably, the between-group differences in the SII were statistically significant using either SMDs, ORs, or HRs. Furthermore, the capacity of the SII to discriminate between the two patient groups was considered good, with an overall AUC value of 0.77. Sensitivity analysis confirmed the stability of the results of the meta-analysis. In meta-regression, the effect size was significantly associated with some markers of inflammation (e.g., albumin and lactate dehydrogenase) but not others (e.g., C-reactive protein and ferritin). Furthermore, no significant associations were observed with known risk factors for adverse outcomes in COVID-19, such as diabetes, hypertension, and cardiovascular disease (75–77). This supports the proposition that the SII may provide additional information regarding the extent of systemic inflammation in COVID-19 and could therefore be helpful in enhancing the capacity to identify those patients at risk of severe disease or death early after hospital admission. Furthermore, the lack of significant associations between the effect size and the year of publication suggests that the capacity of the SII to discriminate between patients with severe disease/non-survivor status and patients with non-severe disease/survivor status was maintained through the first three years of the COVID-19 pandemic and was not influenced by different vaccination status and vaccine dose, new SARS-CoV-2 variants, and the progressive introduction of anti-inflammatory and immunomodulatory treatment strategies. Interestingly, subgroup analysis identified differences in effect size associated with the specific study continent, particularly for studies reporting ORs and HRs, indicating a possible role of ethnicity in influencing the association between the SII and COVID-19.

The SII was initially investigated in 2014 as an inflammatory biomarker to predict clinical outcomes in cancer patients (20). Since then, an increasing number of studies, including systematic reviews and meta-analyses, have reported that the SII is significantly associated with a reduced overall survival and/or progression-free survival in several types of cancer, e.g., carcinoma patients receiving immune checkpoint inhibitors (78), prostate cancer (79), gynaecological and breast cancer (80), lung cancer (81), urinary tract cancer (82, 83), and several cancers of the gastrointestinal tract (84–87). Furthermore, relatively high SII values at baseline have been shown to be significantly associated with adverse clinical outcomes in other disease states characterised by a systemic pro-inflammatory state, e.g., stroke (88), non-alcoholic fatty liver disease (89), and ischaemic heart disease (90). Notably, in patients with coronary heart disease undergoing revascularisation the predictive capacity of the SII for adverse clinical outcomes was superior to that of traditional cardiovascular risk factors, further supporting the potential clinical utility of this index in routine practice (91). The COVID-19 pandemic has provided further impetus to investigate the potential clinical use of the SII, given the established role of a state of excess systemic inflammation in the pathophysiology of the disease and the related risk of complications and adverse outcomes (92, 93). Compared to other indexes investigated in patients with COVID-19 that are derived from routine haematological parameters, e.g., NLR and the platelet-to-lymphocyte ratio (PLR), the SII captures information from three key blood cell types involved in the pathophysiology of inflammation, i.e., neutrophils, platelets, and lymphocytes. The potential superiority of the SII over other indices in COVID-19 was initially reported by Fois et al. (47). In their study, multivariate analysis showed that the SII, but not other indices such as the aggregate index of systemic inflammation, NLR, monocyte-to-lymphocyte ratio, PLR, and systemic inflammation response index, was independently associated with the primary endpoint, hospital mortality (47).

The comprehensive appraisal of the published literature in our study provides robust evidence of the potential clinical utility of the SII in COVID-19 patients. This is further supported by the observed pooled AUC value and the results of the Fagan’s nomogram, which indicated that the post-test probability of severe disease/mortality was significantly different from the pre-test probability (94). However, before its introduction in clinical practice, further research is warranted to justify the use of the SII for the routine assessment of patients with COVID-19. In particular, appropriately designed prospective studies should investigate the predictive capacity of the SII singly or in combination with other biomarkers of inflammation and to determine the influence of specific patient characteristics. For example, in addition to the potential effects of ethnicity previously described, recent epidemiological studies have reported that age and sex can also influence the SII (95, 96).

The observed moderate-substantial between-study heterogeneity is a significant limitation of our study. However, subgroup analysis identified specific sources of heterogeneity when the effect size was expressed as SMD (study endpoint), OR (study continent), and HR (study continent). Furthermore, there was significant publication bias in studies reporting the OR whereas no assessment could be performed in studies reporting the HR because of their limited number. As discussed, a significant strength of our systematic review and meta-analysis was the comprehensive assessment of the clinical significance of the SII by means of meta-regression and subgroup analysis, SROC, and Fagan’s nomogram.

In conclusion, our systematic review and meta-analysis has shown that higher SII values on admission are significantly associated with severe clinical manifestations and the risk of mortality in hospitalized COVID-19 patients. Further prospective studies are warranted to determine whether this haematologically derived inflammatory biomarker can further enhance, singly or in combination with other inflammatory, demographic, or clinical parameters, the prognosis and management of a wide range of COVID-19 patient populations, including patients of different ethnicity.

## Data availability statement

The original contributions presented in the study are included in the article/**Supplementary Material**. Further inquiries can be directed to the corresponding author.

## Author contributions

Study conception: AZ, AM. Data collection and analysis: AZ. Data interpretation: AZ, AM. Writing - first draft: AM. Writing - Review and Editing, AZ, AM. All authors contributed to the article and approved the submitted version.

## Supplementary material

Supplementary Figure 1 Sensitivity analysis of the association between the SII and COVID-19. The middle vertical axis indicates the overall standardized mean difference, and the two vertical axes indicate the 95% confidence intervals. The hollow circles represent the pooled standardized mean difference when the remaining study is omitted from the meta-analysis. The two ends of each broken line represent the 95% confidence intervals.

Supplementary Figure 2 Funnel plot of studies reporting SII values in COVID-19 patients with different disease severity and survival status after “trimming-and-filling.” Dummy studies and genuine studies are represented by enclosed circles and free circles, respectively.

Supplementary Figure 3 Forest plot of studies examining the SII in patients with COVID-19 according to specific endpoint.

Supplementary Figure 4 Forest plot of studies examining SII in patients with COVID-19 according to specific geographic area.

Supplementary Figure 5 Sensitivity analysis of the association between the SII and COVID-19 disease. The influence of individual studies on the overall odds ratio (OR) is shown. The middle vertical axis indicates the overall OR and the two vertical axes indicate the 95% confidence intervals (CI). Hollow circles represent the pooled OR when the remaining study is omitted from the meta-analysis. The two ends of each broken line represent the 95% CI.

Supplementary Figure 6 Forest plot of studies examining the association between the SII and disease severity or survival status in patients with COVID-19 by means of odds ratio, after removing the studies of Xue et al and Cocos et al (44, 72).

Supplementary Figure 7 Forest plot of studies examining the SII in patients with COVID-19 by means of odds ratio, according to specific endpoint.

Supplementary Figure 8 Forest plot of studies examining the SII in patients with COVID-19 by means of odds ratio, according to the study continent.

Supplementary Figure 9 Sensitivity analysis of the association between SII and COVID-19 disease using hazard ratio (HR). The middle vertical axis indicates the overall HR, and the two vertical axes indicate the 95% confidence intervals (CI). The hollow circles represent the pooled HR when the remaining study is omitted from the meta-analysis. The two ends of each broken line represent the 95% CI.

Supplementary Figure 10 Forest plot of studies examining the SII in patients with COVID-19 by means of hazard ratio, according to the study continent.

Supplementary Figure 11 Deeks’ funnel plot asymmetry test for the assessment of publication bias.

Supplementary Figure 12 The bivariate boxplot exploring heterogeneity across the studies.

Supplementary Figure 13 Forest plots of sensitivity and specificity for the study level covariates included in the univariate meta-regression model.
