# Supplementary figures and images for: Systemic inflammation index, disease severity, and mortality in patients with COVID-19: a systematic review and meta-analysis
Source: Front Immunol. 2023 Jun 21;14:1212998. doi: 10.3389/fimmu.2023.1212998 (PMC10320859; doi:10.3389/fimmu.2023.1212998)

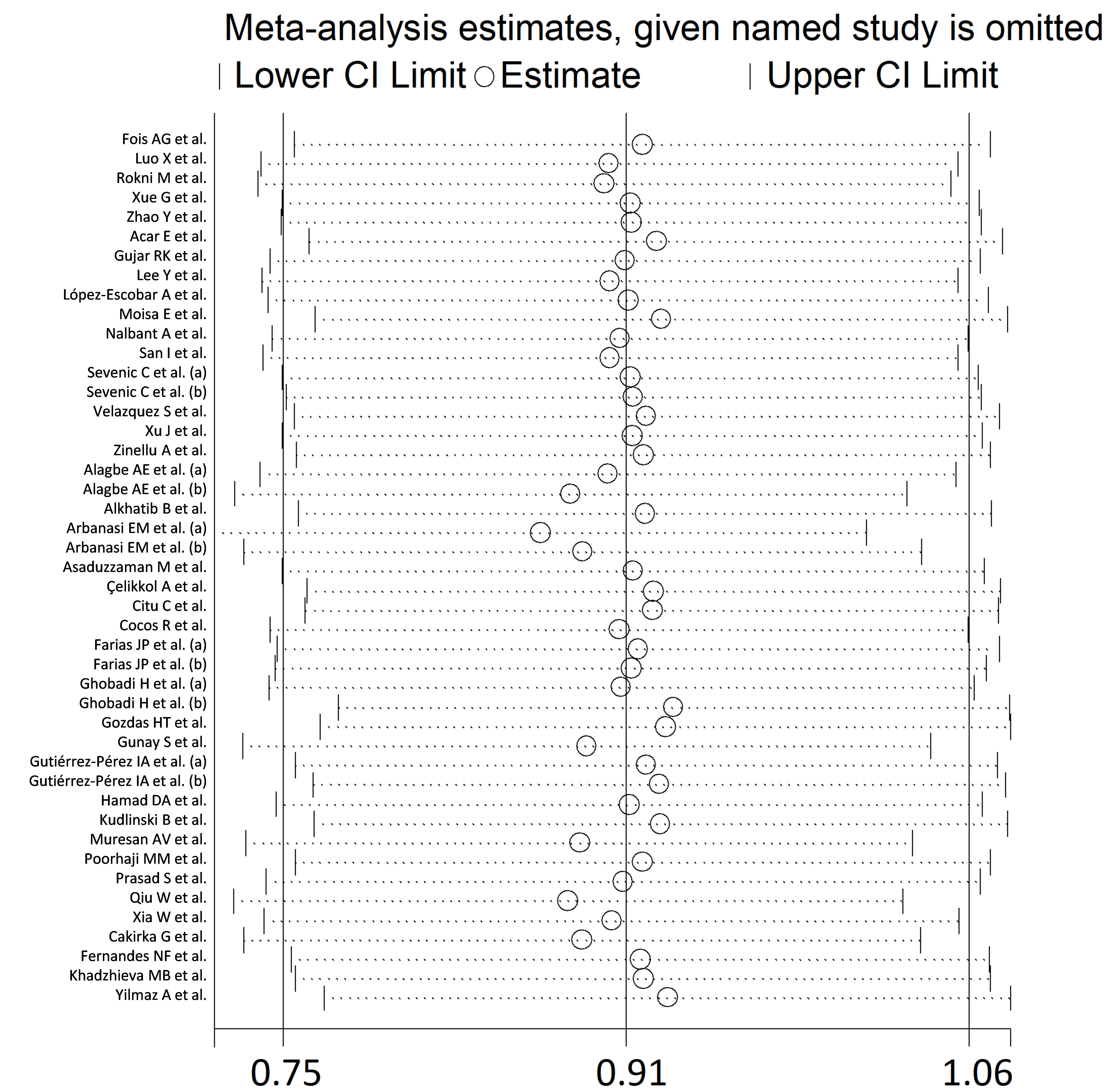

Supplement: Supplementary Figure 1 — Sensitivity analysis of the association between the SII and COVID-19. The middle vertical axis indicates the overall standardized mean difference, and the two vertical axes indicate the 95% confidence intervals. The hollow circles represent the pooled standardized mean difference when the remaining study is omitted from the meta-analysis. The two ends of each broken line represent the 95% confidence intervals. [file Image_1.tif]

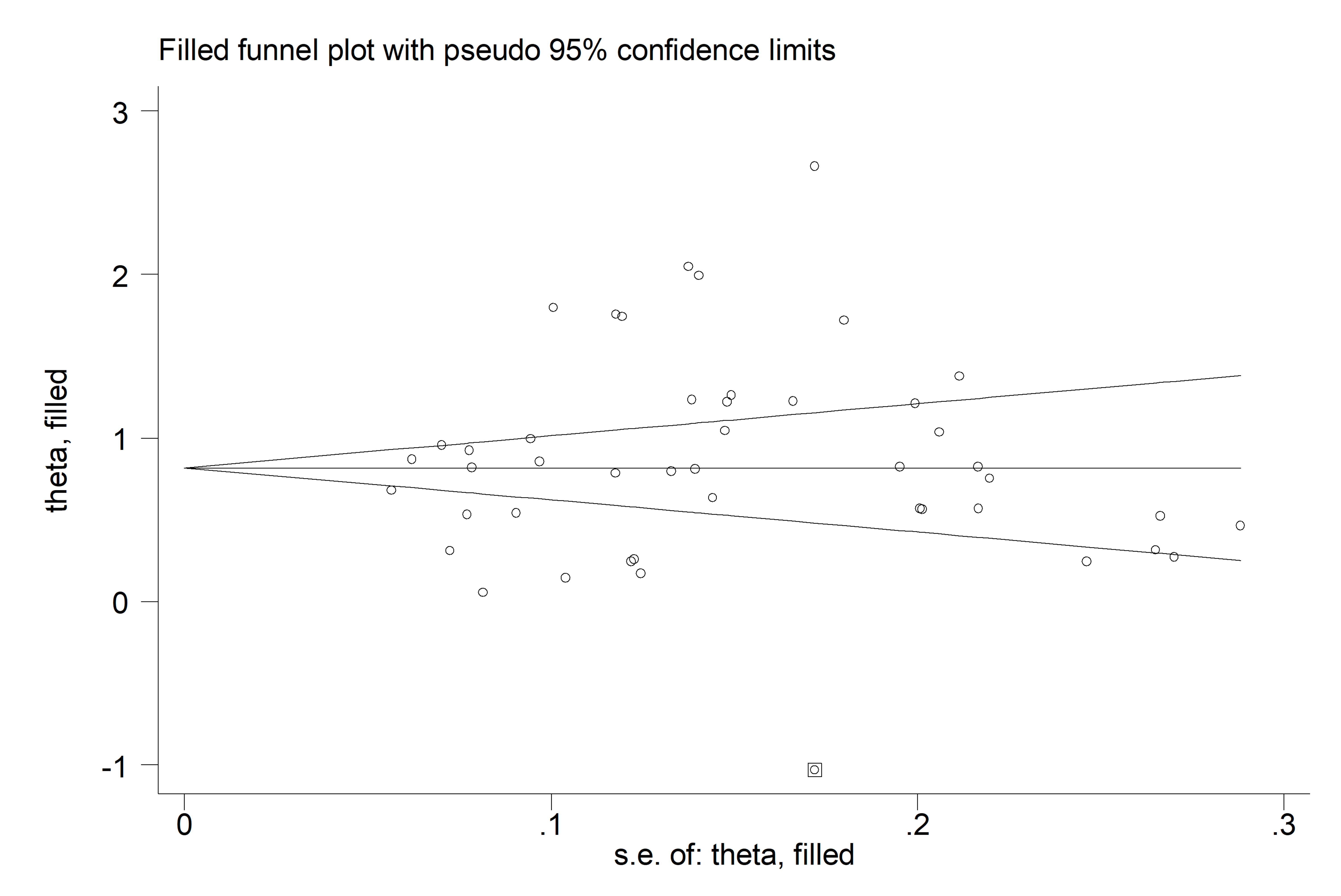

Supplement: Supplementary Figure 2 — Funnel plot of studies reporting SII values in COVID-19 patients with different disease severity and survival status after “trimming-and-filling.” Dummy studies and genuine studies are represented by enclosed circles and free circles, respectively. [file Image_2.tif]

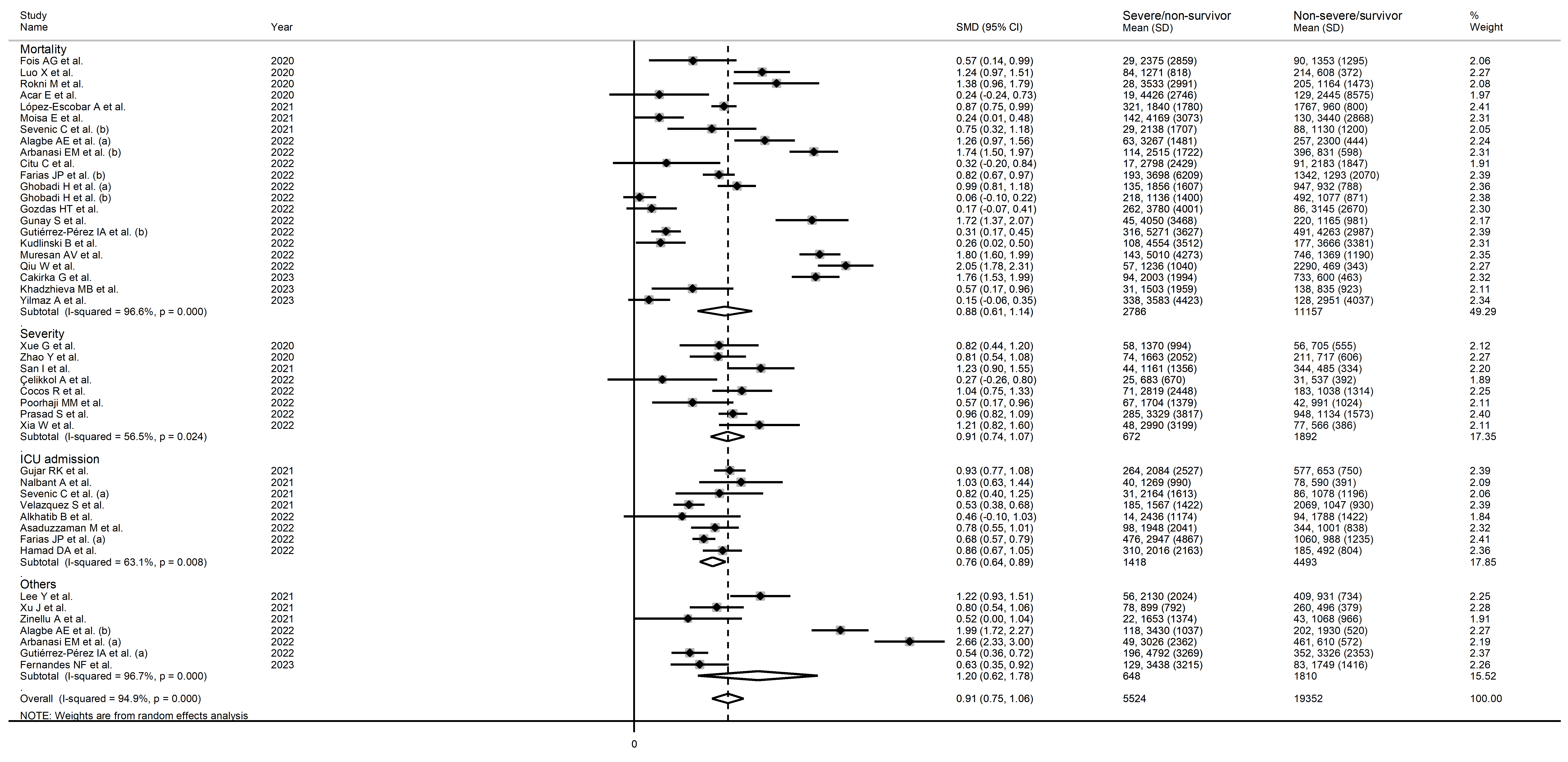

Supplement: Supplementary Figure 3 — Forest plot of studies examining the SII in patients with COVID-19 according to specific endpoint. [file Image_3.tif]

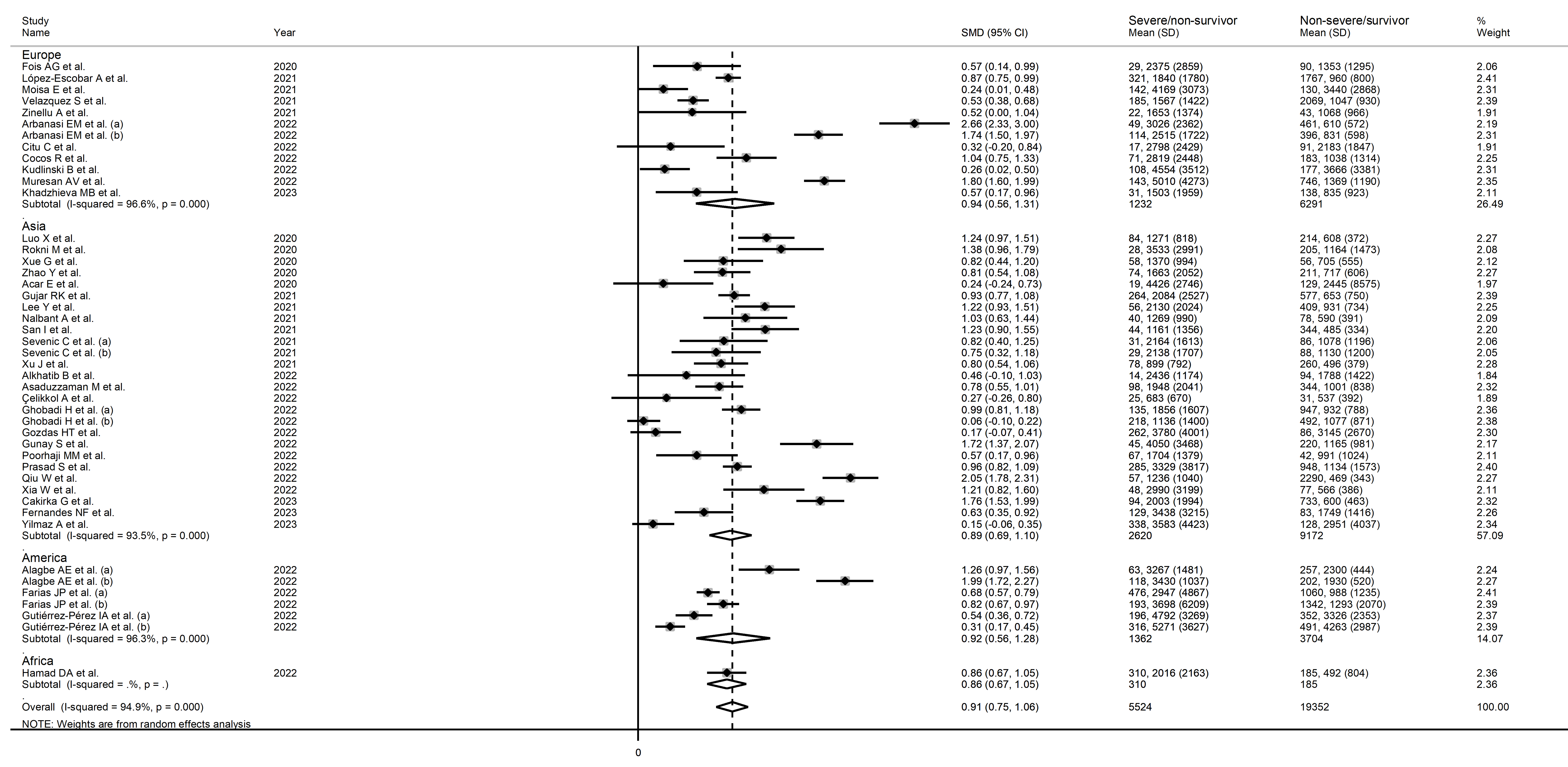

Supplement: Supplementary Figure 4 — Forest plot of studies examining SII in patients with COVID-19 according to specific geographic area. [file Image_4.tif]

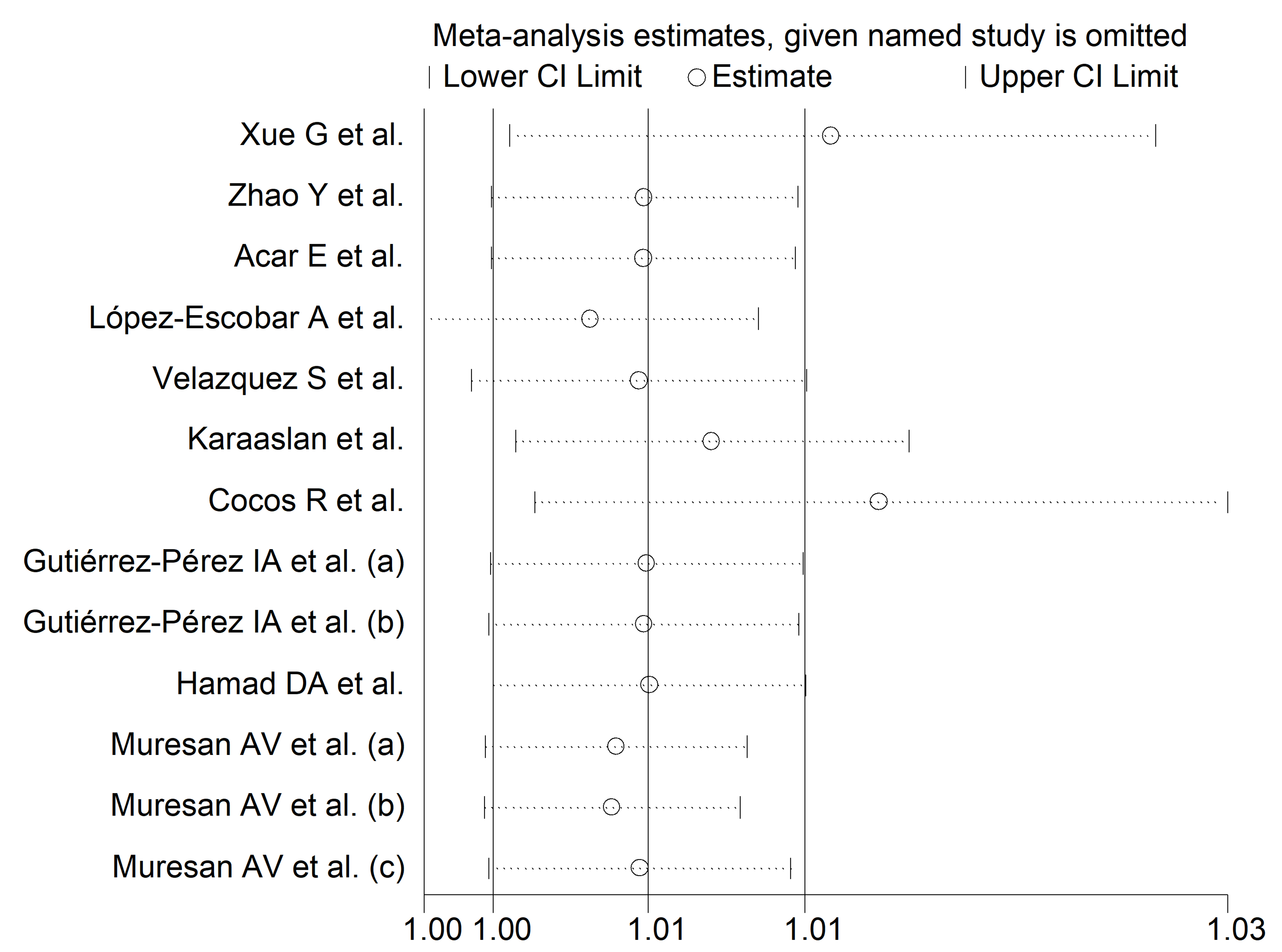

Supplement: Supplementary Figure 5 — Sensitivity analysis of the association between the SII and COVID-19 disease. The influence of individual studies on the overall odds ratio (OR) is shown. The middle vertical axis indicates the overall OR and the two vertical axes indicate the 95% confidence intervals (CI). Hollow circles represent the pooled OR when the remaining study is omitted from the meta-analysis. The two ends of each broken line represent the 95% CI. [file Image_5.tif]

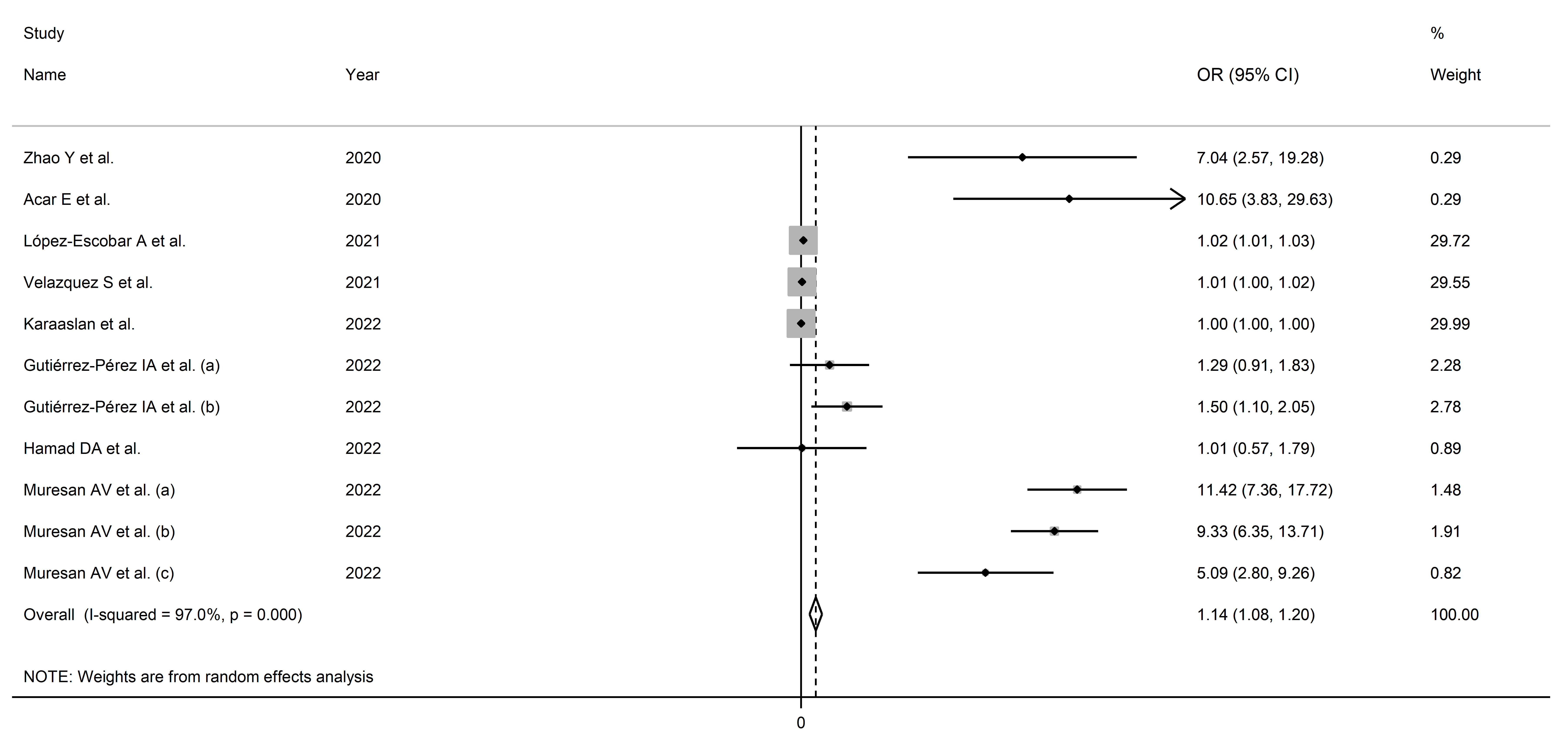

Supplement: Supplementary Figure 6 — Forest plot of studies examining the association between the SII and disease severity or survival status in patients with COVID-19 by means of odds ratio, after removing the studies of Xue et al and Cocos et al (44, 72). [file Image_6.tif]

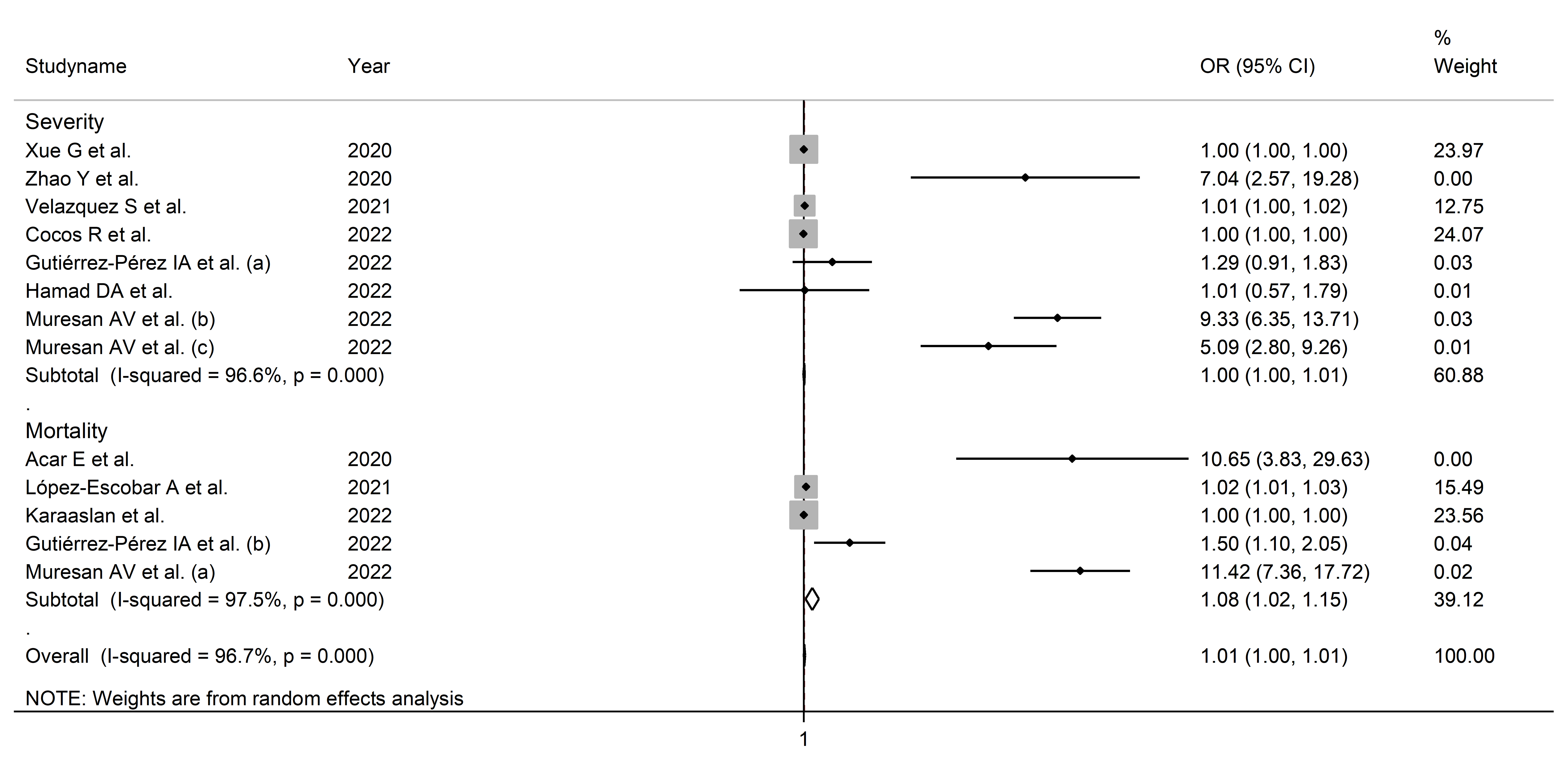

Supplement: Supplementary Figure 7 — Forest plot of studies examining the SII in patients with COVID-19 by means of odds ratio, according to specific endpoint. [file Image_7.tif]

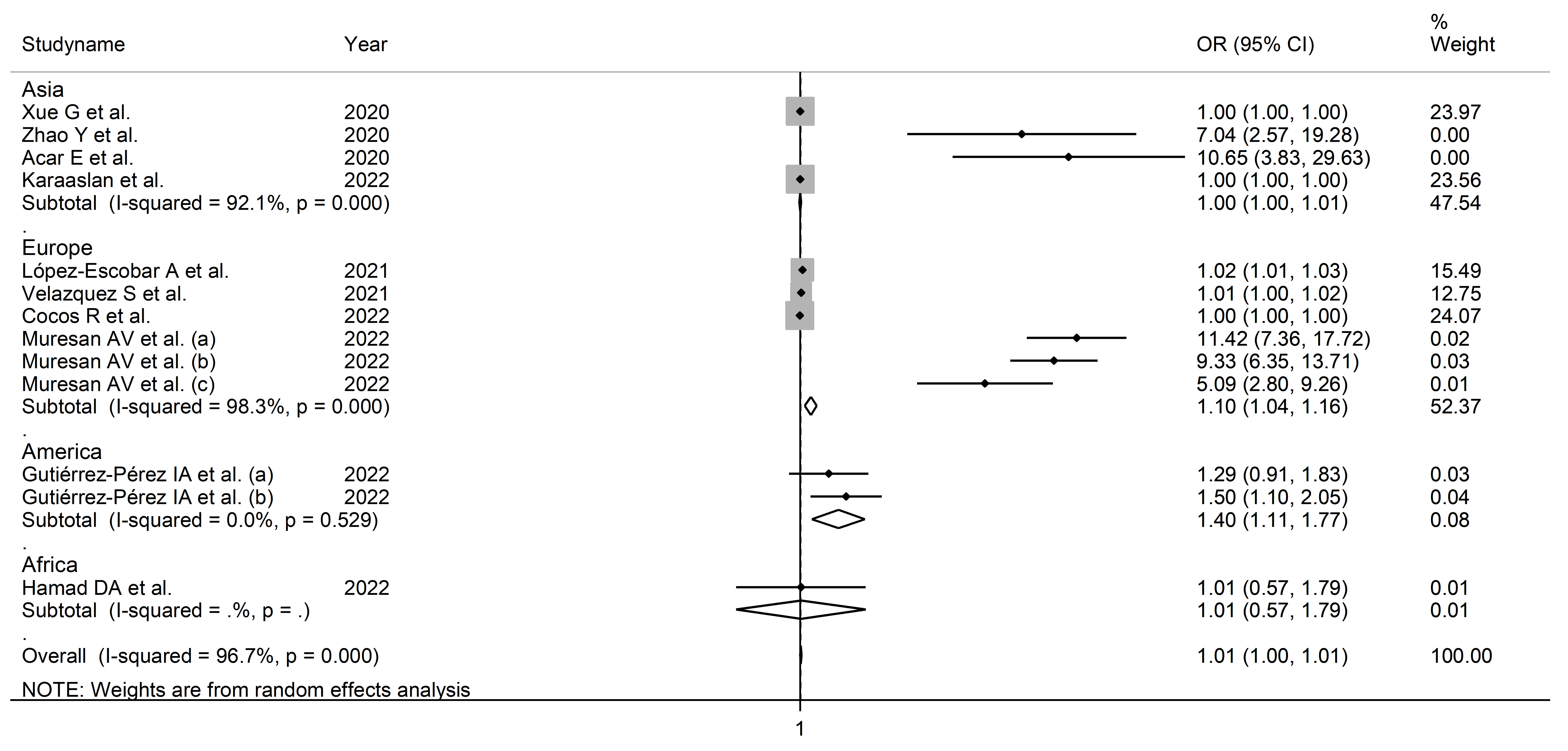

Supplement: Supplementary Figure 8 — Forest plot of studies examining the SII in patients with COVID-19 by means of odds ratio, according to the study continent. [file Image_8.tif]

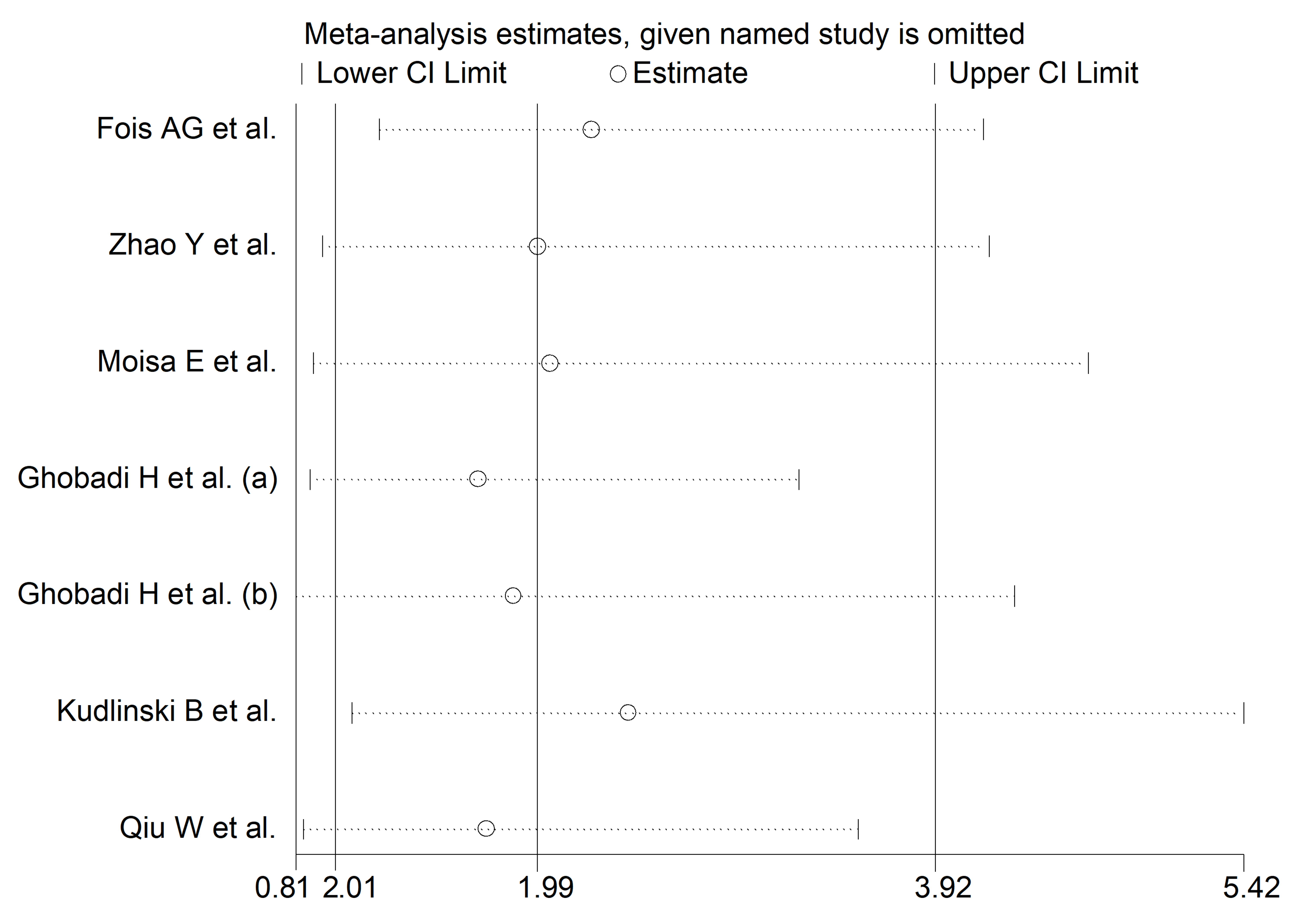

Supplement: Supplementary Figure 9 — Sensitivity analysis of the association between SII and COVID-19 disease using hazard ratio (HR). The middle vertical axis indicates the overall HR, and the two vertical axes indicate the 95% confidence intervals (CI). The hollow circles represent the pooled HR when the remaining study is omitted from the meta-analysis. The two ends of each broken line represent the 95% CI. [file Image_9.tif]

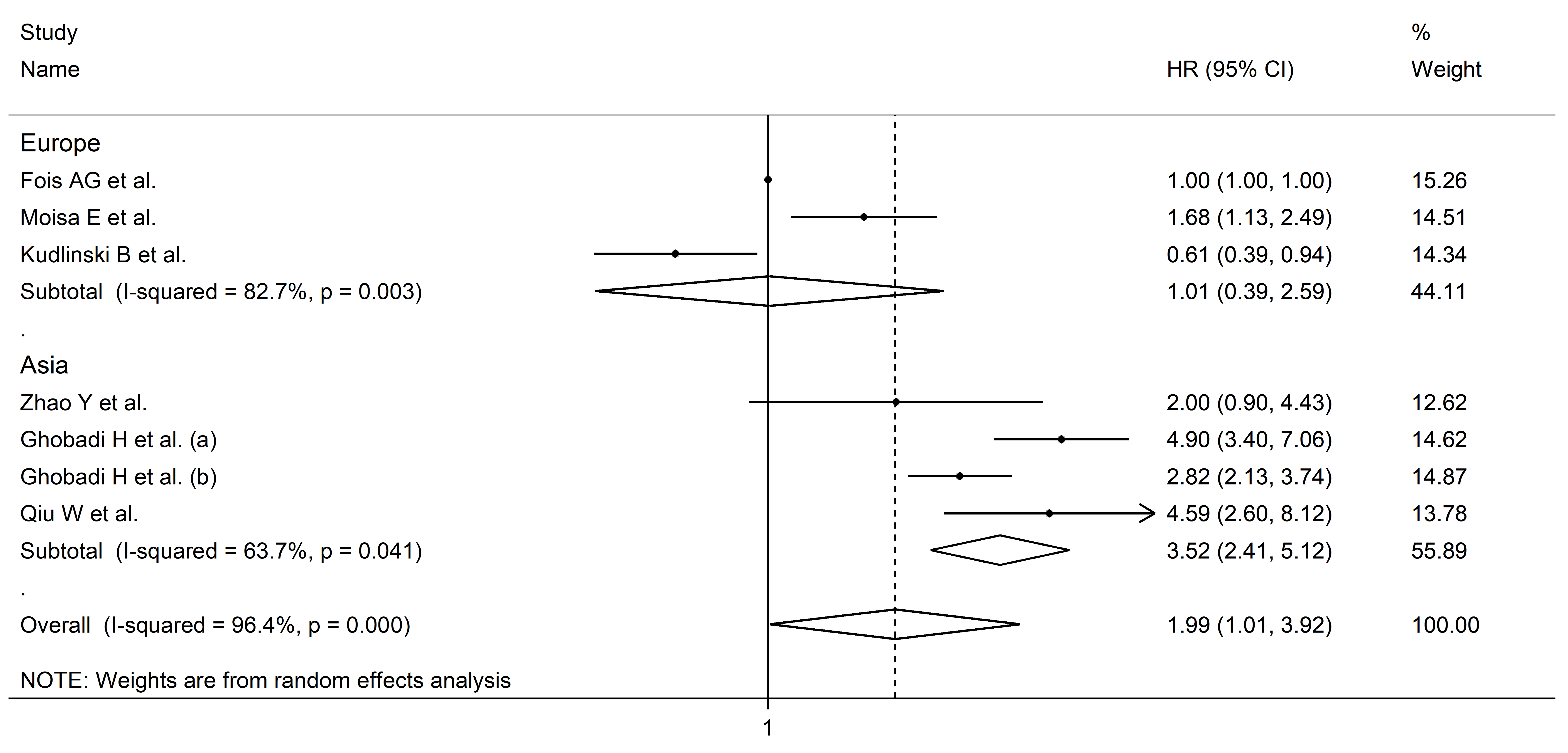

Supplement: Supplementary Figure 10 — Forest plot of studies examining the SII in patients with COVID-19 by means of hazard ratio, according to the study continent. [file Image_10.tif]

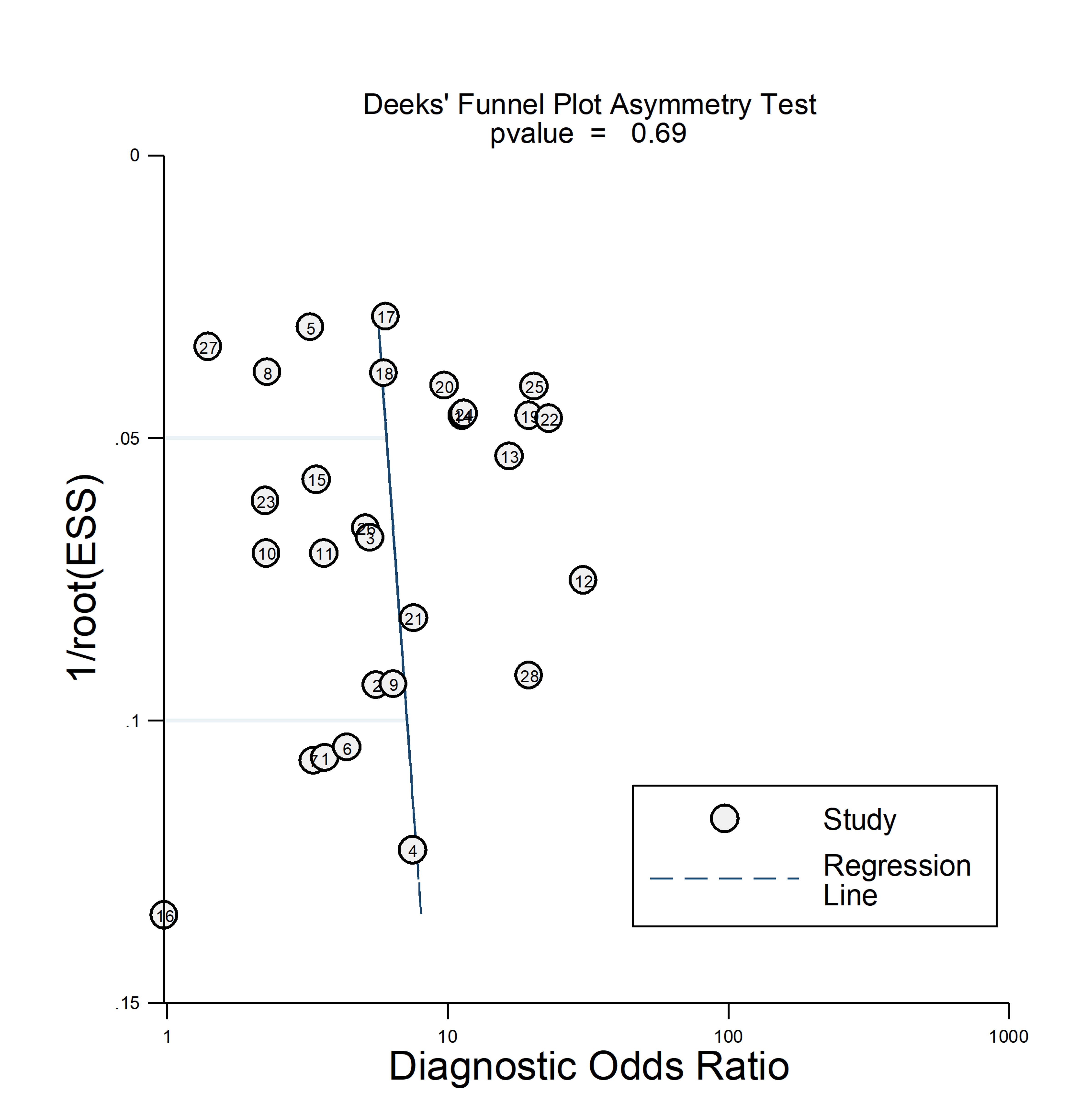

Supplement: Supplementary Figure 11 — Deeks’ funnel plot asymmetry test for the assessment of publication bias. [file Image_11.tif]

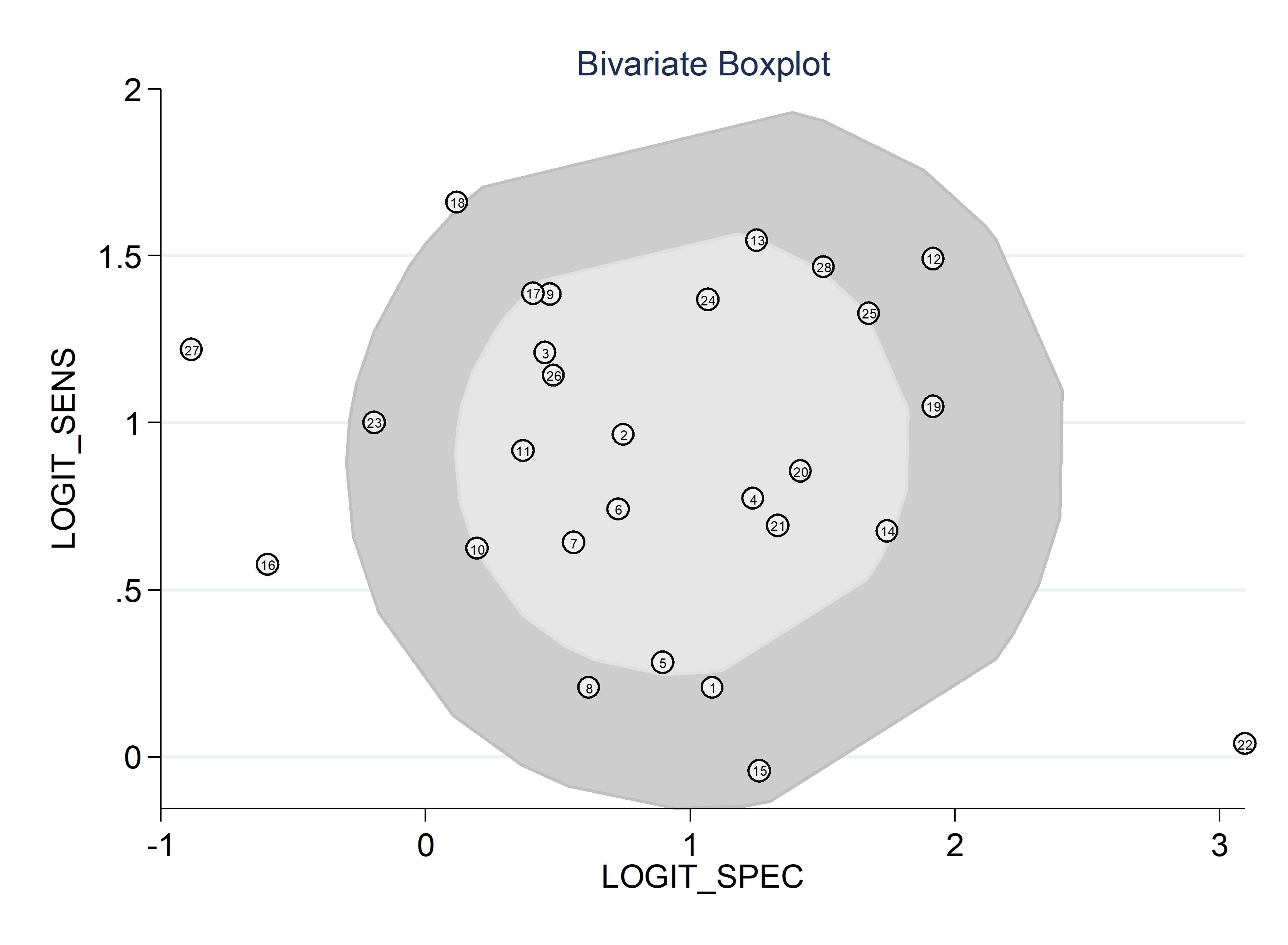

Supplement: Supplementary Figure 12 — The bivariate boxplot exploring heterogeneity across the studies. [file Image_12.tif]

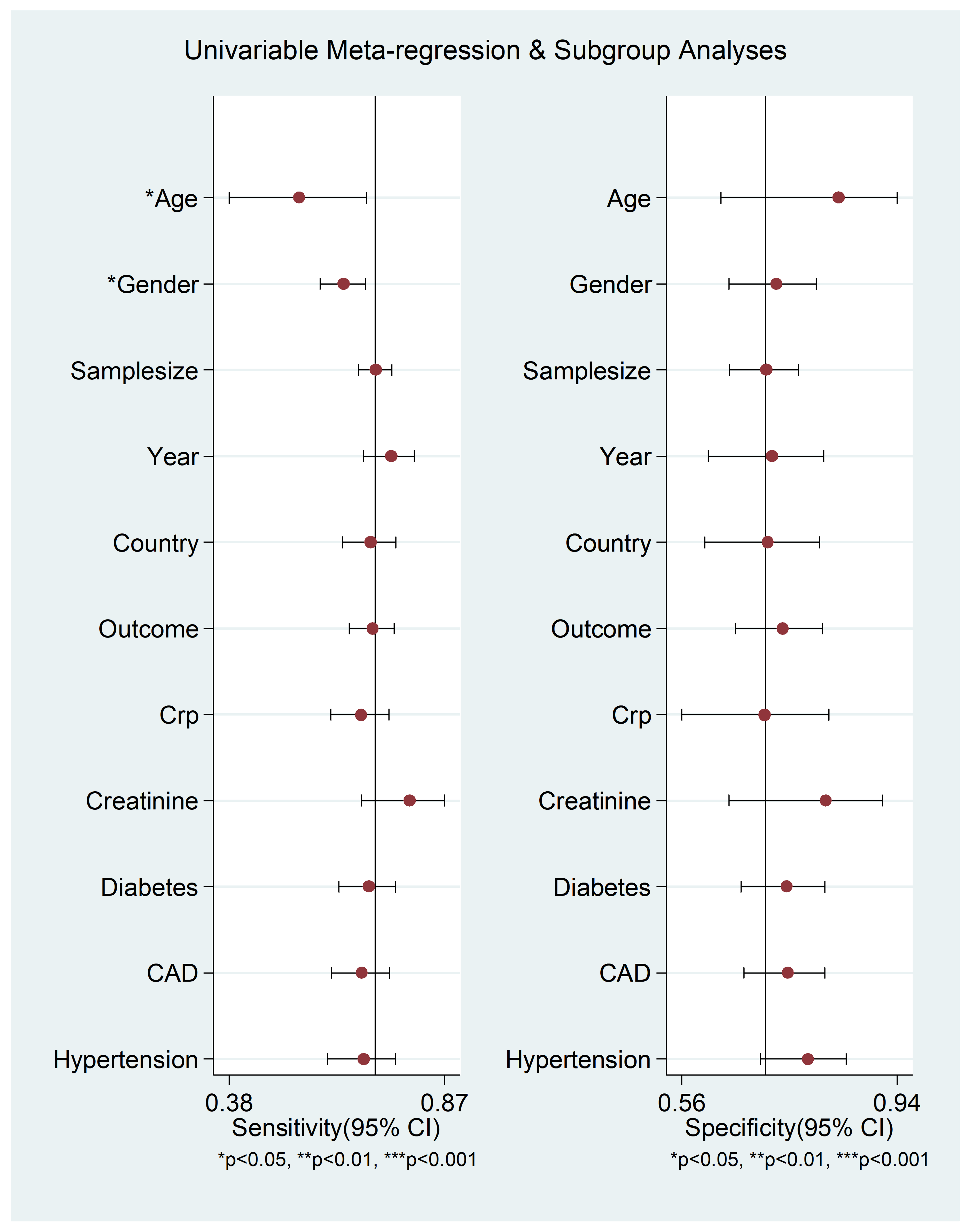

Supplement: Supplementary Figure 13 — Forest plots of sensitivity and specificity for the study level covariates included in the univariate meta-regression model. [file Image_13.tif]
