# Supplementary material for: Systemic inflammation index, disease severity, and mortality in patients with COVID-19: a systematic review and meta-analysis
Source: Front Immunol. 2023 Jun 21;14:1212998. doi: 10.3389/fimmu.2023.1212998 (PMC10320859; doi:10.3389/fimmu.2023.1212998)
Supplement: Supplementary file 16 [file Table_3.docx]

**Supplementary Table 3.** The Joanna Briggs Institute critical appraisal checklist.

| **Study** | **Were the criteria for inclusion clearly defined?** | **Were the subjects and the setting described in detail?** | **Was the exposure measured in a valid and reliable way?** | **Were objective, standard criteria used for measurement of the condition?** | **Were confounding factors identified?** | **Were strategies to deal with confounding factors stated?** | **Were the outcomes measured in a valid and reliable way?** | **Was appropriate statistical analysis used?** | **Risk of bias** |
| --- | --- | --- | --- | --- | --- | --- | --- | --- | --- |
| Fois AG et al (47) | Yes | Yes | Yes | Yes | Yes | Yes | Yes | Yes | Low |
| Luo X et al (59) | Yes | Yes | Yes | Yes | Yes | Yes | Yes | Yes | Low |
| Rokni M et al (66) | Yes | Yes | Yes | Yes | No | No | Yes | No | High |
| Xue G et al (72) | Yes | Yes | Yes | Yes | Yes | Yes | Yes | Yes | Low |
| Zhao Y et al (74) | Yes | Yes | Yes | Yes | Yes | Yes | Yes | Yes | Low |
| Acar E et al (36) | Yes | Yes | Yes | Yes | Yes | Yes | Yes | Yes | Low |
| Gujar RK et al (50) | Yes | Yes | Yes | Yes | No | No | Yes | No | High |
| Li Y et al (57) | Yes | Yes | Yes | Yes | Yes | Yes | Yes | Yes | Low |
| López-Escobar A et al (58) | Yes | Yes | Yes | Yes | Yes | Yes | Yes | Yes | Low |
| Moisa E et al (60) | Yes | Yes | Yes | Yes | Yes | Yes | Yes | Yes | Low |
| Nalbant A et al (62) | Yes | Yes | Yes | Yes | No | No | Yes | No | High |
| San I et al (67) | Yes | Yes | Yes | Yes | Yes | Yes | Yes | Yes | Low |
| Sevinc C et al (68) | Yes | Yes | Yes | Yes | No | No | Yes | No | High |
| Velazquez S et al (69) | Yes | Yes | Yes | Yes | Yes | Yes | Yes | Yes | Low |
| Xu J et al (71) | Yes | Yes | Yes | Yes | Yes | Yes | Yes | Yes | Low |
| Zinellu A et al (21) | Yes | Yes | Yes | Yes | Yes | Yes | Yes | Yes | Low |
| Alagbe AE et al (37) | Yes | Yes | Yes | Yes | Yes | Yes | Yes | Yes | Low |
| Alkhatib B et al (38) | Yes | Yes | Yes | Yes | No | No | Yes | No | High |
| Arbanasi EM et al (39) | Yes | Yes | Yes | Yes | Yes | Yes | Yes | Yes | Low |
| Asaduzzaman M et al (40) | Yes | Yes | Yes | Yes | Yes | Yes | Yes | Yes | Low |
| Çelikkol A et al (42) | Yes | Yes | Yes | Yes | No | No | Yes | No | High |
| Citu C et al (43) | Yes | Yes | Yes | Yes | Yes | Yes | Yes | Yes | Low |
| Cocos R et al (44) | Yes | Yes | Yes | Yes | Yes | Yes | Yes | Yes | Low |
| Farias JP et al (45) | Yes | Yes | Yes | Yes | Yes | Yes | Yes | Yes | Low |
| Ghobadi H et al (48) | Yes | Yes | Yes | Yes | No | No | Yes | No | High |
| Gozdas HT et al (49) | Yes | Yes | Yes | Yes | Yes | Yes | Yes | Yes | Low |
| Gunay S et al (51) | Yes | Yes | Yes | Yes | Yes | Yes | Yes | Yes | Low |
| Gutiérrez-Pérez IA et al (52) | Yes | Yes | Yes | Yes | Yes | Yes | Yes | Yes | Low |
| Hamad DA et al (53) | Yes | Yes | Yes | Yes | Yes | Yes | Yes | Yes | Low |
| Karaaslan et al (54) | Yes | Yes | Yes | Yes | Yes | Yes | Yes | Yes | Low |
| Kudlinski B et al (56) | Yes | Yes | Yes | Yes | Yes | Yes | Yes | Yes | Low |
| Muresan AV et al (61) | Yes | Yes | Yes | Yes | Yes | Yes | Yes | Yes | Low |
| Poorhaji MM et al (63) | Yes | Yes | Yes | Yes | No | No | Yes | No | High |
| Prasad S et al (64) | Yes | Yes | Yes | Yes | Yes | Yes | Yes | Yes | Low |
| Qiu W et al (65) | Yes | Yes | Yes | Yes | Yes | Yes | Yes | Yes | Low |
| Xia W et al (70) | Yes | Yes | Yes | Yes | No | No | Yes | No | High |
| Cakirka G et al (41) | Yes | Yes | Yes | Yes | Yes | Yes | Yes | Yes | Low |
| Fernandes NF et al (46) | Yes | Yes | Yes | Yes | No | No | Yes | No | High |
| Khadzhieva MB et al (55) | Yes | Yes | Yes | Yes | Yes | Yes | Yes | Yes | Low |
| Yilmaz A et al (73) | Yes | Yes | Yes | Yes | Yes | Yes | Yes | Yes | Low |
